# Synthesis of aliphatic α-hydroxy carboxylic acids *via* electrocarboxylation of aldehydes

**DOI:** 10.1039/d5ra07885g

**Published:** 2025-11-21

**Authors:** Valtteri Oksanen, Kiia Malinen, Tom Wirtanen

**Affiliations:** a Chemical and Polymer Synthesis, VTT Technical Research Centre of Finland Ltd, Box 1000 FI-02044 Espoo Finland tom.wirtanen@vtt.fi tom.wirtanen@iki.fi; b School of Chemical Engineering, Aalto University Box 11000 FI-00076 Aalto Finland

## Abstract

Herein, we describe electrocarboxylation of aliphatic aldehydes in an undivided cell for the synthesis of aliphatic α-hydroxy acids (AHA). The electrosynthesis utilizes stable carbon-based electrodes providing safe and simple access to AHAs. Yields between 20 to 45% are acheived with seven substrates, some up to three times higher than with previous electrosynthesis protocols.

## Abstract 2

A novel method for synthesis of aliphatic α-hydroxy acids is achieved *via* electrocarboxylation of aldehydes with CO_2_ in an undivided cell with stable carbon based electrodes.

## Graphical abstract

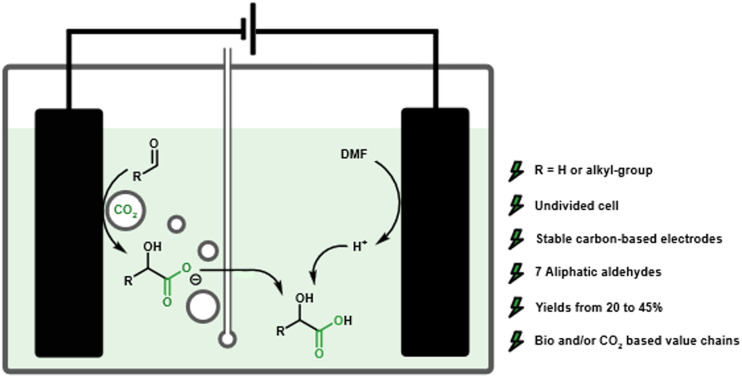

## Introduction

The α-hydroxy acids (AHA) are compounds with significant applications in the chemical, cosmetics, and pharmaceutical industries.^1^ For example, glycolic and lactic acids are used as monomers,^1^ and mandelic acids are used in pharmaceuticals and antibacterial applications.^2,3^ Furthermore, AHAs are also valuable intermediates for other chemicals such as α-keto acids,^4,5^ α-hydroxyamides,^6^ and α-amino acids.^5,7^

**Scheme 1:**
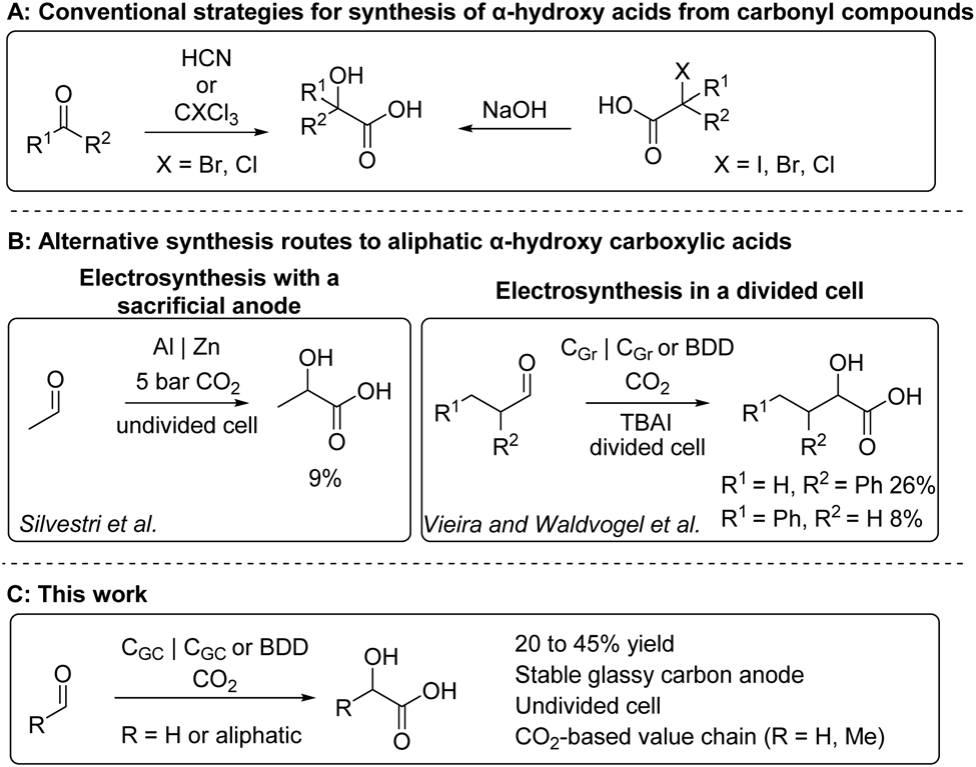
(A) Conventional approaches for synthesis of hydroxy acids from carbonyl compounds. (B) Synthetic methods for aliphatic α-hydroxy acids and scope of aliphatic products. (C) This work; electrosynthesis of aliphatic α-hydroxy acids in an undivided cell with stable carbon-based electrodes.

AHAs are commonly prepared by hydrogenolysis of cyanohydrin or α-halocarboxylic acid intermediates, which are synthesized from carbonyl compounds using highly toxic cyanides or halogens, respectively (Scheme 1A).^1^ Alternatively, chloroform and bromoform can be used as C_1_-synthons.^8,9^ Hence, more sustainable and less hazardous synthesis routes are preferable. Ideally, CO_2_ could be employed as a sustainable and non-toxic C_1_-synthon, while its use, especially in high-volume chemicals such as monomers, could contribute towards significant utilization of CO_2_, especially when aldehydes are also prepared from carbon dioxide.^10,11^

Electrochemical transformations remain some of the most prominent ways for converting CO_2_ into value-added products either *via* direct reduction of CO_2_ or generation of high-energy intermediates that are able to react with CO_2_.^12–15^ Particularly, the latter modus operandi is intriguing as it allows the efficient synthesis of carboxylic acids and their derivatives from a broad scope of functional groups such as olefins,^16,17^ organic (pseudo)halides,^17,18^ carbonyls^17,19^ and arenes.^20^

One of the main advantages of electrosynthesis is the possibility to apply electrons and electron holes as reductants and oxidants instead of stoichiometric reagents, potentially reducing the amount of waste generated and by providing safe and controllable access to reactive radical intermediates.^21–23^ Given the recent interest in electrosynthesis, it is rather surprising that there are only two studies that describe the synthesis of a total of three different aliphatic α-hydroxy acids from aldehydes (Scheme 1B). Silvestri and co-workers reported the synthesis of lactic acid using a sacrificial aluminium anode in an undivided cell under 5 bar CO_2_ pressure,^24^ whereas Vieira & Waldvogel and co-workers reported synthesis of 2-hydroxy-4-phenyl-butyric and 2-hydroxy-3-phenylbutyric acids in a divided cell with stable carbon-based electrodes.^25^ Besides these electrosynthesis, aliphatic aldehydes have been seldomly utilized as starting materials in carboxylations with other methods further underlining the need for new protocols.^26^

We became interested in the electrocarboxylation of aliphatic aldehydes as the relative production rates of α-hydroxy acids and side products, alcohols and vicinal diols, remains largely unexplored. Furthermore, expanding the carboxylation scope and increasing the yields could enable the synthesis of various fully bio- and CO_2_-based α-hydroxy carboxylic acids, for example, for polymer applications. Noteworthy, only one report has previously demonstrated the electrosynthesis of aromatic α-hydroxy acids in an undivided cell with stable anodes.^27^

## Results and discussion

**Fig. 1:**
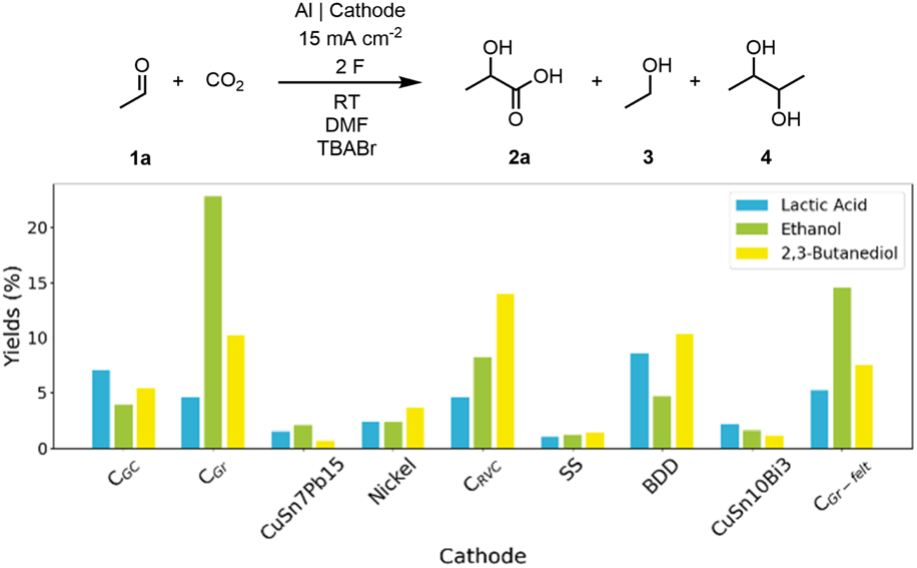
Yields of different electroreduction products by cathode material.

To ensure the industrial relevance we conducted all the experiments in 20 mL undivided cells under galvanostatic conditions, which provides a simple and robust design for scale-up.^28^ We began our study by using acetaldehyde (1a) as our model compound as it has been shown to undergo reductive electrocarboxylation under 5 bar CO_2_.^24^ First, we studied the performance of various cathode materials for the reduction of 0.1 M 1a in 0.02 M TBABr DMF solution at 15 mA cm^−2^ under constant CO_2_ bubbling using an aluminium anode. Gratifyingly, all the cathodes provided lactic acid (2a), although the yields were below 10% (Fig. 1). Generally, carbon-based electrodes gave higher yields for 2a, ethanol (3) and 2,3-butanediol (4) when compared to metal electrodes CuSn7Pb15, Ni, stainless steel (SS, AISI 316) and CuSn10Bi3, which could be due to carbon-based electrodes low electrocatalytic activity towards CO_2_ reduction.^29^ The highest yields for lactic acid were obtained with boron-doped diamond (BDD, 9%) and glassy carbon (C_GC_, 7%). Interestingly, 3 was obtained as the main product with graphite (C_Gr_, 23%) and graphite felt (C_Gr-felt_, 15%) cathodes whereas the highest yield for 4 was received with reticulated vitreous carbon (C_RVC_, 14%). We also screened different aprotic solvents able to dissolve CO_2_ such MeCN, propylene carbonate (PC), and *N*-butyl-2-pyrrolidone (NBP) (Table S2) with sacrificial Al anode. No lactic acid was detected with PC and NBP whereas MeCN yielded 3% of lactic acid.

Sacrificial anodes are problematic due to the generation of stoichiometric amounts of metal waste, altering of the inter-electrode distance, and passivation during the electrolysis.^30^ Therefore, we wanted to develop reaction conditions for stable carbon-based anodes (C_Gr_ and C_GC_). We opted to perform further optimizations with C_GC_ as the cathode since it gave the highest selectivity for the lactic acid in the initial trials (Fig. 1). When acetonitrile and C_Gr_ anode were used, ethanol (5%) was the only reduction product in acetonitrile (Table 1 Entry 1).

Next, we moved to investigate binary solvent mixtures of MeCN and DMF (Table 1, Entries 2–4). Interestingly, when equivolume ratio of MeCN and DMF was employed, lactic acid 2a was obtained with both C_Gr_ and C_GC_ anodes in 19 and 21% yield, respectively (Entries 2 and 4). Furthermore, 2a was obtained in 14% yield when the MeCN : DMF ratio was lowered to 9 : 1 with C_Gr_ anode (Entry 3). We also investigated if temperature-dependant CO_2_ solubility^31^ could accentuate carboxylation with C_GC_ anode. On the contrary, 2a yield remained practically same when the reaction was performed at 0 °C (19%, Entry 5) and 40 °C (18%, Entry 6).

**Table 1:** Optimization of synthesis of α-hydroxy acids *via* electroreduction of aldehydes under continuous carbon dioxide bubbling^a^

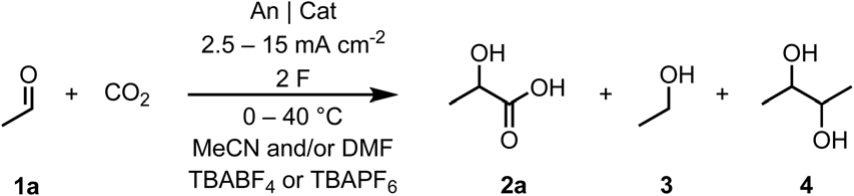						
Entry	MeCN : DMF ratio	c.d. (mA cm^−2^)	X^−^	*T* (°C)	An|Cat	Yield (%) (2a : 3 : 4)
1	1 : 0	15	BF_4_	RT	C_Gr_|C_GC_	0 : 5 : 0
2	1 : 1	15	BF_4_	RT	C_Gr_|C_GC_	19 : 8 : 12
3	9 : 1	15	BF_4_	RT	C_Gr_|C_GC_	14 : 5 : 14
4	1 : 1	15	BF_4_	RT	C_GC_|C_GC_	21 : 9 : 21
5	1 : 1	15	BF_4_	0	C_GC_|C_GC_	19 : 11 : 29
6	1 : 1	15	BF_4_	40	C_GC_|C_GC_	18 : 8 : 15
7	1 : 1	5	BF_4_	RT	C_GC_|C_GC_	20 : 7 : 6
8	1 : 1	5	PF_6_	RT	C_GC_|C_GC_	20 : 7 : 7
9	0 : 1	5	PF_6_	RT	C_GC_|C_GC_	27 : 4 : 4
10	0 : 1	2.5	PF_6_	RT	C_GC_|C_GC_	10 : 6 : 9
**11**	**0 : 1**	**5**	**PF**_**6**_	**RT**	**C**_**GC**_ **|BDD**	**28 : 6 : 9**
12	0 : 1	10	PF_6_	RT	C_GC_|C_GC_	22 : 12 : 20

a Yields determined by ^1^H NMR using 1,3,5-trimethoxybenzene (TMB) as an internal standard.

Next, we investigated decreasing current density from 15 mA cm^−2^ to 5 mA cm^−2^ (Entry 7). This modification also decreased 2,3-butanediol yield from 21% (Entry 4) to 6% (Entry 7). However, the yield of 2a remained practically unchanged (21% in Entry 4 *vs.* 20% in Entry 7). Thereafter, we continued to investigate the effect of the supporting electrolyte anion. The yields of all three products were comparable with both TBABF_4_ and TBAPF_6_ electrolytes (Entries 7 and 8). Finally, when the reaction was performed in sole DMF with current density of 5 mA cm^−2^, 2a yield increased to 27% (Entry 9). Interestingly, decreasing the current density further to 2.5 mA cm^−2^ led into diminished 2a yield of 10% (Entry 10). The optimized reaction conditions function also with BDD as the cathode (Entry 11), which gave a minor increase in 2a yield (28%) compared to C_GC_ (Entry 9). Furthermore, 2,3-butanediol and ethanol yields increased from 4% to 6% and from 4% to 9%, respectively. When the current density from Entry 9 was increased to 10 mA cm^−2^ in DMF, the yield of 2a decreased to 22% demonstrating that the increased yield was not solely due to the different solvent system (Entry 12).

Further optimization studies (current density, solvent, additives, supporting electrolyte) with a BDD cathode did not improve the yields. Thus, we performed several control experiments for uncovering additional details of the synthesis (Table S6). The conversion of the acetaldehyde 1a was in all cases near 100%, which was higher than the combined yields of the reaction products. Interestingly, when the reaction was conducted in the absence of current, 48% conversion was recorded, indicating that the continuous bubbling of CO_2_ can evaporate 1a from the cell (Table S6, Entry 2). The use of CO_2_ pre-saturated solution reduced the conversion of 1a from 97% (Entry S1) to 68% (Entry S3). However, the yield of the lactic acid 2a was also reduced to 14%. In addition, when the reaction was performed using anhydrous DMF (Entry S6), the yield of 2a significantly decreased to 5% (*vide infra*), indicating that residual water is necessary for higher carboxylation yields. The importance of water was investigated further with additional control experiments. Water concentration of 0.08 vol% resulted in slightly diminished 2a yield of 25% (Table S6, Entry 7), whereas increasing it to 0.32 vol% water provided practically identical yield in comparison to optimized conditions (27%, Entry S8). Interestingly, when the water concentration was increased even further to 0.64 vol%, lactic acid yield was marginally improved to 30%, whereas yields of ethanol (10%) and 2,3-butanediol (18%) also increased in comparison to optimized conditions (Entry S9). Finally, we studied how the applied charge affects the current efficiency (c.e.). Interestingly, when 0.2 F charge was passed, c.e. of 2a was increased from 28% (Entry S1) to 36% (Entry S4). Concurrently, the c.e. of the side products were diminished from 6 to 4% and 9 to 7% for ethanol and 2,3-butanediol, respectively. The concentration of the aldehyde is also an important parameter, and when Entry S5 was repeated with 10-fold concentration of 0.5 M, using the same current density and 0.2 F charge, the c.e. of 2a was only 9% (Entry S5), accompanied by with significantly increased c.e. of 2,3-butanediol and ethanol to 60% and 17%, respectively.

In order to explain the decrease in c.e. from 0.2 F to 2 F, we hypothesized that lactic acid might decompose by anodic oxidation. To investigate this, we employed cyclic voltammetry with C_GC_ working electrode (SI) and observed that the lactic acid is not oxidized in DMF. However, sodium lactate shows an anodic peak at 0.64 V (*vs.* Fc/Fc^+^). Finally, we performed the same measurement from the reaction mixture after electro-carboxylation. We did not observe any lactate oxidation by CV from the mixture, indicating that the formed lactate is (partially) protonated during the electrolysis. We hypothesize that only the protonated fraction is protected from the anodic degradation *viz.* the unprotonated fraction might be oxidized at the anode. Inspired by these findings, we studied the effect of additives for the counter-oxidation (*e.g.*, TEA, TBAI, TBD) and for the protonation of the lactate (HCOOH), but these approaches did not improve the lactic acid yield further (SI).

We also performed cyclic voltammetry with the same set-up to investigate the cathodic reduction mechanism (SI). Carbon dioxide reduction was not observed in the employed potential window (0.01 V–−2.49 V *vs.* Fc/Fc^+^). Conversely, two consecutive cathodic peaks at −1.27 V and −1.34 V (*vs.* Fc/Fc^+^) were observed for acetaldehyde in Ar saturated DMF, alongside peak at −1.24 V during the return scan. However, when acetaldehyde is reduced in CO_2_ saturated DMF, CV curve is altered. The first cathodic peak is retained at −1.27 V and the second reduction peak disappears. Furthermore, there is no oxidative peak in the return scan, indicating that radical anion generated in the first reduction reacts rapidly with CO_2_, which implies towards EC mechanism.

**Scheme 2:**
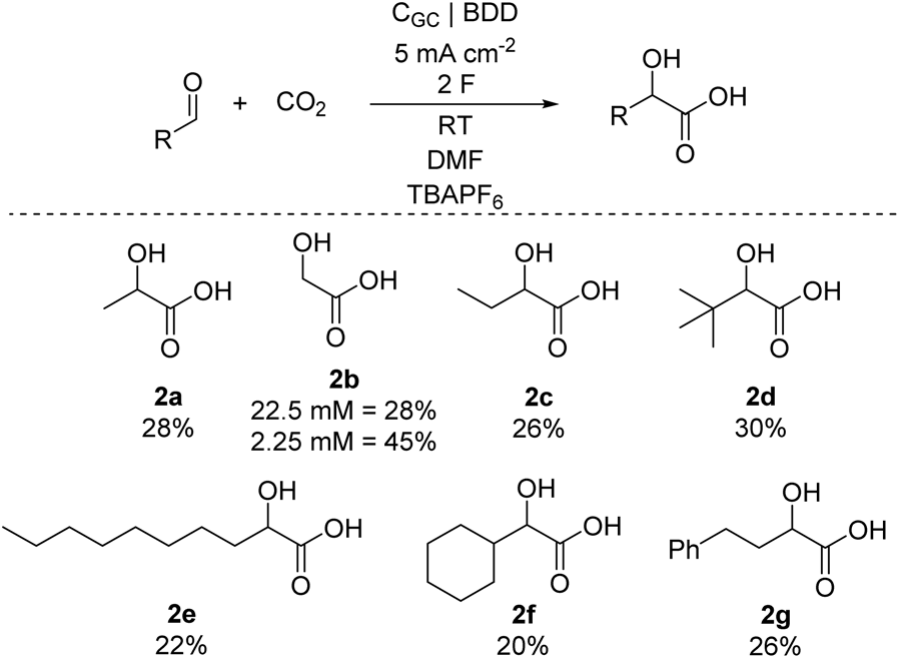
Yields of different α-hydroxy acids of electrocarboxylation of 50 mM aldehyde solutions under continuous CO_2_ bubbling.

The applicability of the reaction was then investigated with other aliphatic aldehydes (Scheme 2). First, aldehydes were carboxylated using optimized conditions (Table 1, Entry 11), and after recording ^1^H NMR based yield, AHAs were isolated as their corresponding benzyl esters (SI, Section 7) by adding triethylamine and benzyl bromide to the electrolysis cell and mixing the solution for two hours at 70 °C. We explored whether increasing the sterics at β-carbon is tolerated by using propanal, 2,2-dimethylpropanal, and cyclohexanecarboxaldehyde as starting materials. In general, these compounds reacted similarly to acetaldehyde and provided α-hydroxyacids **2c**, **2d** and 2f in 26%, 30%, and 20% yields, respectively. We then investigated using formaldehyde as a starting material for the synthesis of glycolic acid 2b, which is an emerging monomer. Due to technical reasons (SI), the reaction was performed in different concentrations. Interestingly, 2.25 mM formaldehyde solution yielded 45% of 2b, while 28% of 2b was obtained with 22.5 mM solution. Furthermore, nonanal was also suitable coupling partner for CO_2_ furnishing 22% of 2e. Moreover, the reaction is tolerant to phenyl group as a γ-substituent in respect to the aldehyde, demonstrated by the carboxylation of 3-phenylpropionaldehyde into 2g in 26% yield. Conversely, ethyl glyoxylate and *trans*-2-butenoic acid did not yield corresponding hydroxy acids under standard conditions. Preliminary studies on carboxylation of aromatic aldehydes resulted in yields below 10%, indicating that reaction conditions should be optimized independently for this compound class. Furthermore, the tolerance towards different functional groups was also examined. Generally, amides (DMF, Table 1), nitriles (MeCN, Table 1) and alcohols (IPA, Table S8) are well tolerated. Furthermore, carboxylic acids (formic acid, Table S3), HFIP (Table S8) and aryl halides (PhCl, PhI, Table S8) resulted in reduced AHA yields. Conversely, presence of amine (TEA, Table S3) or guanidine (TBD, Table S3) inhibited carboxylation almost completely whereas anhydrides (Ac_2_O, Table S8), metal salts with low reduction potential (ZnCl_2_, Table S8), alkynediols (1,4-butynediol, Table S8) and silyl halides (TMSCl, Table S8) resulted in a total inhibition of the reaction.

**Scheme 3:**
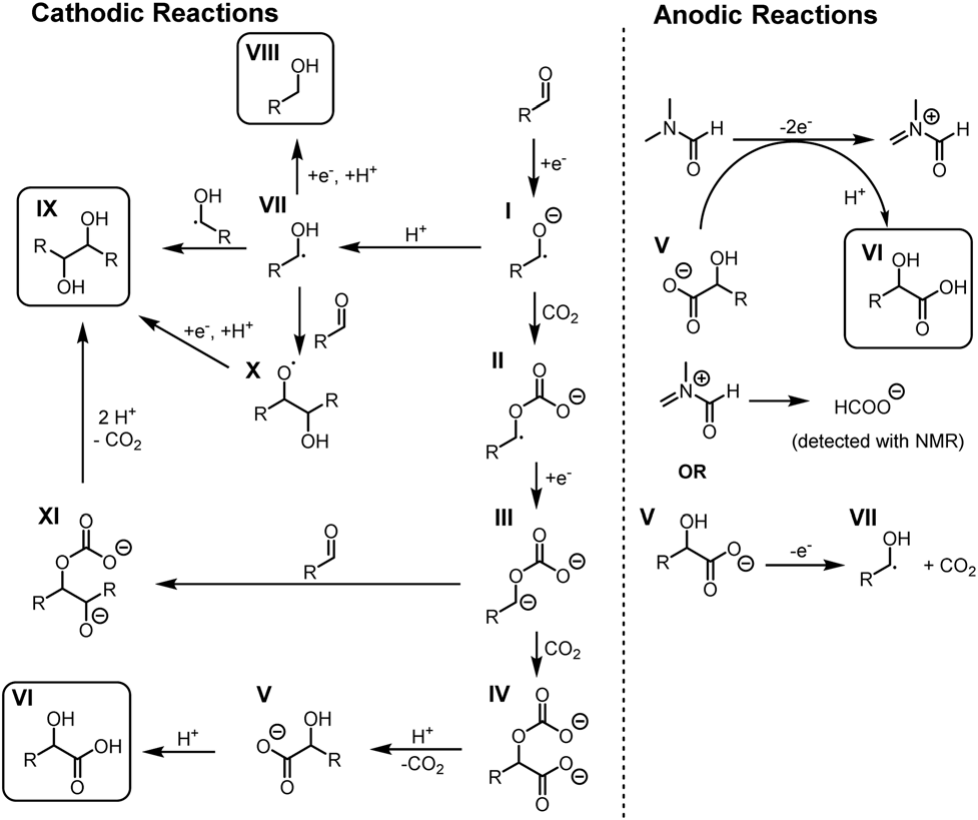
Plausible mechanism for electrocarboxylation in undivided cell.

Based on the control studies, cyclic voltammetry and previous studies,^25,27,32^ we propose following mechanism for electrocarboxylation in undivided cell (Scheme 3). First, aldehyde is reduced to ketyl radical anion I which reacts with carbon dioxide, giving carboxylated radical anion II. The reduction of II gives dianion III which reacts with carbon dioxide, giving double carboxylated dianion IV. Decarboxylative protonation of IV yields α-hydroxy carboxylate V. Based on the voltametric studies, V is either protonated to α-hydroxy acid VI in wet DMF, enabled by solvent oxidation^33^ and water facilitated proton transfer, or oxidized into hydroxyalkyl radical VII in anhydrous conditions.

We also employed radical trapping experiments by adding CHANT radical trap into electrolysis.^34^ ESI-HRMS analysis showed mass corresponding to CHANT adduct of formyloxyl radical (SI), which could be rationalised by either CO_2_ reduction or DMF oxidation. Furthermore, when reaction was performed in a divided cell, formate was detected from anolyte by ^1^H NMR, which indicates that DMF is oxidised into formate.

Formation of side products could be rationalised by protonation of radical anion I into VII, which could be reduced and protonated into corresponding alcohol VIII or dimerise with other VII radical, yielding vicinal diol IX. However, our control experiments indicate that the c.e. for IX formation depends on the concentration of the aldehyde. Thus, IX could form also *via* reaction of VII with aldehyde, forming oxygen centred radical intermediate X, yielding vicinal diol IX after reduction and subsequent protonation. Alternatively, formation of IX could be explained by a nucleophilic addition of dianion III to aldehyde. It has been shown that similar dianions can react with aldehyde, giving intermediate XI which yielded vicinal diols IX after decarboxylative protonation.^35^

## Conclusions

In this work we have demonstrated that it is possible to tune aliphatic aldehyde reactivity towards the production of α-hydroxy acids in an undivided cell. The reaction is applicable to a range of different aliphatic aldehydes with comparable yields regardless of the substituent pattern. Furthermore, the developed method provides up to three times higher yields for aliphatic α-hydroxy acids in comparison to previous electrochemical methods. We anticipate that further research on alternative counter oxidations (*e.g.*, hydrogen oxidation reaction) or additives can be employed to protect α-hydroxy carboxylates from oxidation, improving the yields and enabling synthesis of completely CO_2_ and bio-based AHAs at a larger scale.

## Conflicts of interest

There are no conflicts to declare.

## Supplementary Material

RA-015-D5RA07885G-s001

RA-015-D5RA07885G-s002

## Data Availability

The data supporting this article have been included as part of the supplementary information (SI). Supplementary information: NMR spectra of isolated hydroxy acid benzyl esters as a separate zip file. See DOI: https://doi.org/10.1039/d5ra07885g.
