# Supplementary material for: Synthesis of aliphatic α-hydroxy carboxylic acids via electrocarboxylation of aldehydes
Source: RSC Adv. 2025 Nov 21;15(53):45724–8. doi: 10.1039/d5ra07885g (PMC12637178; doi:10.1039/d5ra07885g)
Supplement: RA-015-D5RA07885G-s001 [file RA-015-D5RA07885G-s001.zip › NMRSpectra_Isolated_products/Phenylmethyl 2-hydroxybutanoate/2/pdata/1/email_VO_LA_S15_final_2_1.pdf]

after column

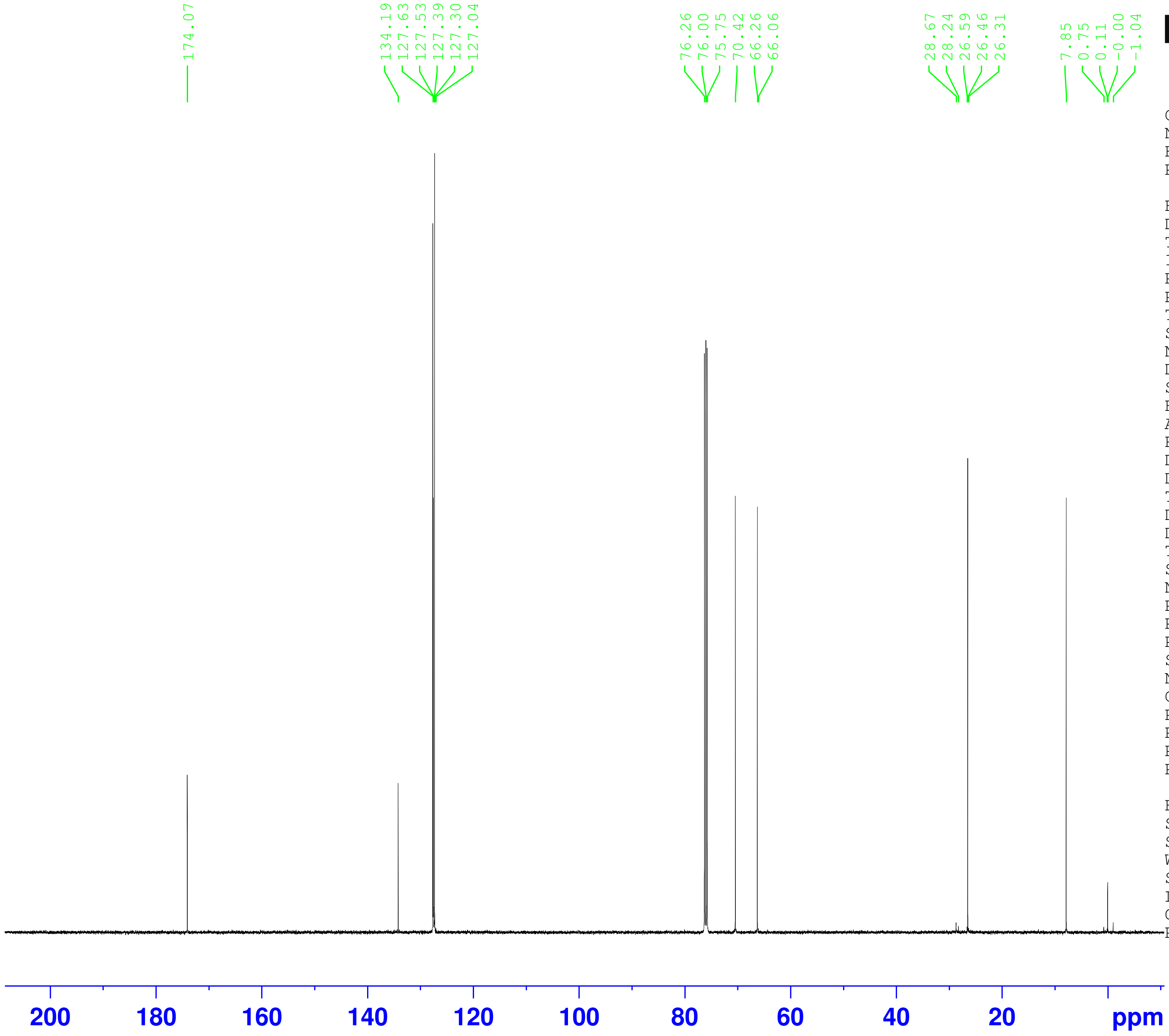

BRUKER

Current Data Parameters

NAME	VO_LA_S15_final	
EXPNO	2	
PROCNO	1	

F2 - Acquisition Parameters

Date_	20250219	
Time	6.50	h
INSTRUM	spect	
PROBHD	Z114229_0012 (	
PULPROG	zgpg30	
TD	65536	
SOLVENT	CDCl3	
NS	1600	
DS	4	
SWH	27573.529	Hz
FIDRES	0.841477	Hz
AQ	1.1883861	sec
RG	191.94	
DW	18.133	usec
DE	6.50	usec
TE	298.0	K
D1	1.50000000	sec
D11	0.03000000	sec
TD0	1	
SFO1	125.7703648	MHz
NUC1	13C	
P0	2.83	usec
P1	8.50	usec
PLW1	114.00000000	W
SFO2	500.1325007	MHz
NUC2	1H	
CPDPRG[2	waltz16	
PCPD2	80.00	usec
PLW2	18.50000000	W
PLW12	0.41624999	W
PLW13	0.20937000	W

F2 - Processing parameters

SI	65536	
SF	125.7579203	MHz
WDW	EM	
SSB	0	
LB	1.00	Hz
GB	0	
PC	1.40	
