# Supplementary material for: Synthesis of aliphatic α-hydroxy carboxylic acids via electrocarboxylation of aldehydes
Source: RSC Adv. 2025 Nov 21;15(53):45724–8. doi: 10.1039/d5ra07885g (PMC12637178; doi:10.1039/d5ra07885g)
Supplement: RA-015-D5RA07885G-s001 [file RA-015-D5RA07885G-s001.zip › NMRSpectra_Isolated_products/Phenylmethyl 2-hydroxydecanoate/1/pdata/1/email_VO_LA_S1_final_1_1.pdf]

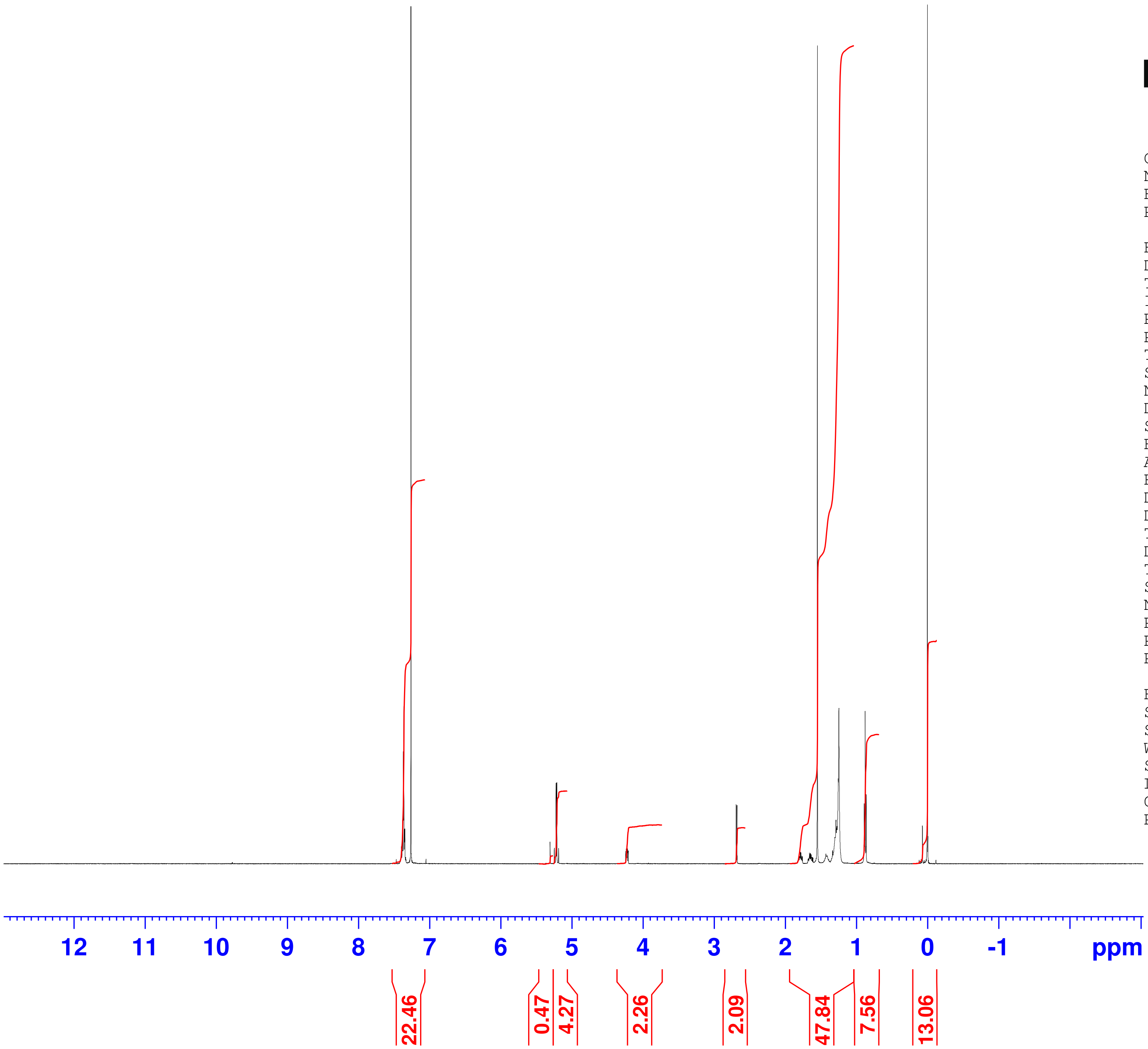

Current Data Parameters

NAME	VO_LA_S1_final
EXPNO	1
PROCNO	1

F2 - Acquisition Parameters

Date_	20241117	
Time	4.23	h
INSTRUM	spect	
PROBHD	Z114229_0012 (	
PULPROG	zg30	
TD	32768	
SOLVENT	CDCl3	
NS	8	
DS	4	
SWH	8012.820	Hz
FIDRES	0.489064	Hz
AQ	2.0447233	sec
RG	191.94	
DW	62.400	usec
DE	17.43	usec
TE	295.1	K
D1	10.00000000	sec
TD0	1	
SFO1	500.1325007	MHz
NUC1	1H	
P0	4.00	usec
P1	12.00	usec
PLW1	18.50000000	W

F2 - Processing parameters

SI	65536	
SF	500.1300115	MHz
WDW	EM	
SSB	0	
LB	0.30	Hz
GB	0	
PC	1.00	
