# Supplementary material for: Synthesis of aliphatic α-hydroxy carboxylic acids via electrocarboxylation of aldehydes
Source: RSC Adv. 2025 Nov 21;15(53):45724–8. doi: 10.1039/d5ra07885g (PMC12637178; doi:10.1039/d5ra07885g)
Supplement: RA-015-D5RA07885G-s001 [file RA-015-D5RA07885G-s001.zip › NMRSpectra_Isolated_products/Phenylmethyl 2-hydroxydecanoate/2/pdata/1/email_VO_LA_S1_final_2_1.pdf]

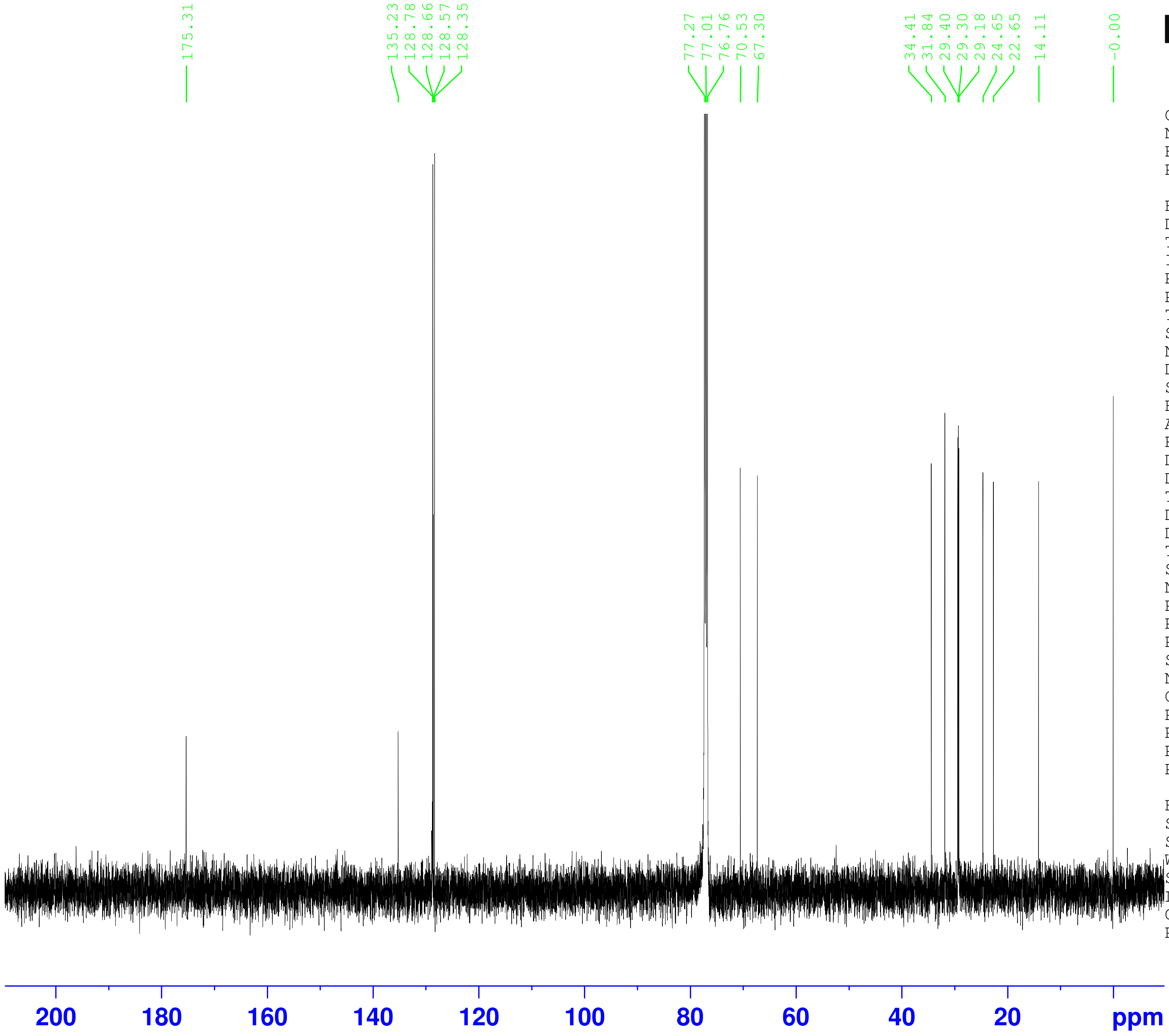

Current Data Parameters

NAME	VO_LA_S1_final
EXPNO	2
PROCNO	1

F2 - Acquisition Parameters

Date_	20241117
Time	10.40 h
INSTRUM	spect
PROBHD	Z114229_0012 (
PULPROG	zgpg30
TD	65536
SOLVENT	CDCl3
NS	8192
DS	4
SWH	27573.529 Hz
FIDRES	0.841477 Hz
AQ	1.1883861 sec
RG	191.94
DW	18.133 usec
DE	6.50 usec
TE	298.0 K
D1	1.50000000 sec
D11	0.03000000 sec
TD0	1
SFO1	125.7703648 MHz
NUC1	13C
P0	2.83 usec
P1	8.50 usec
PLW1	114.00000000 W
SFO2	500.1325007 MHz
NUC2	1H
CPDPRG[2	waltz16
PCPD2	80.00 usec
PLW2	18.50000000 W
PLW12	0.41624999 W
PLW13	0.20937000 W

F2 - Processing parameters

SI	65536
SF	125.7577893 MHz
WDW	EM
SSB	0
LB	1.00 Hz
GB	0
PC	1.40
