# Supplementary material for: Synthesis of aliphatic α-hydroxy carboxylic acids via electrocarboxylation of aldehydes
Source: RSC Adv. 2025 Nov 21;15(53):45724–8. doi: 10.1039/d5ra07885g (PMC12637178; doi:10.1039/d5ra07885g)
Supplement: RA-015-D5RA07885G-s001 [file RA-015-D5RA07885G-s001.zip › NMRSpectra_Isolated_products/Phenylmethyl α-hydroxybenzenebutanoate/6/pdata/1/email_VO_LA_S18_6_1.pdf]

pure isolated

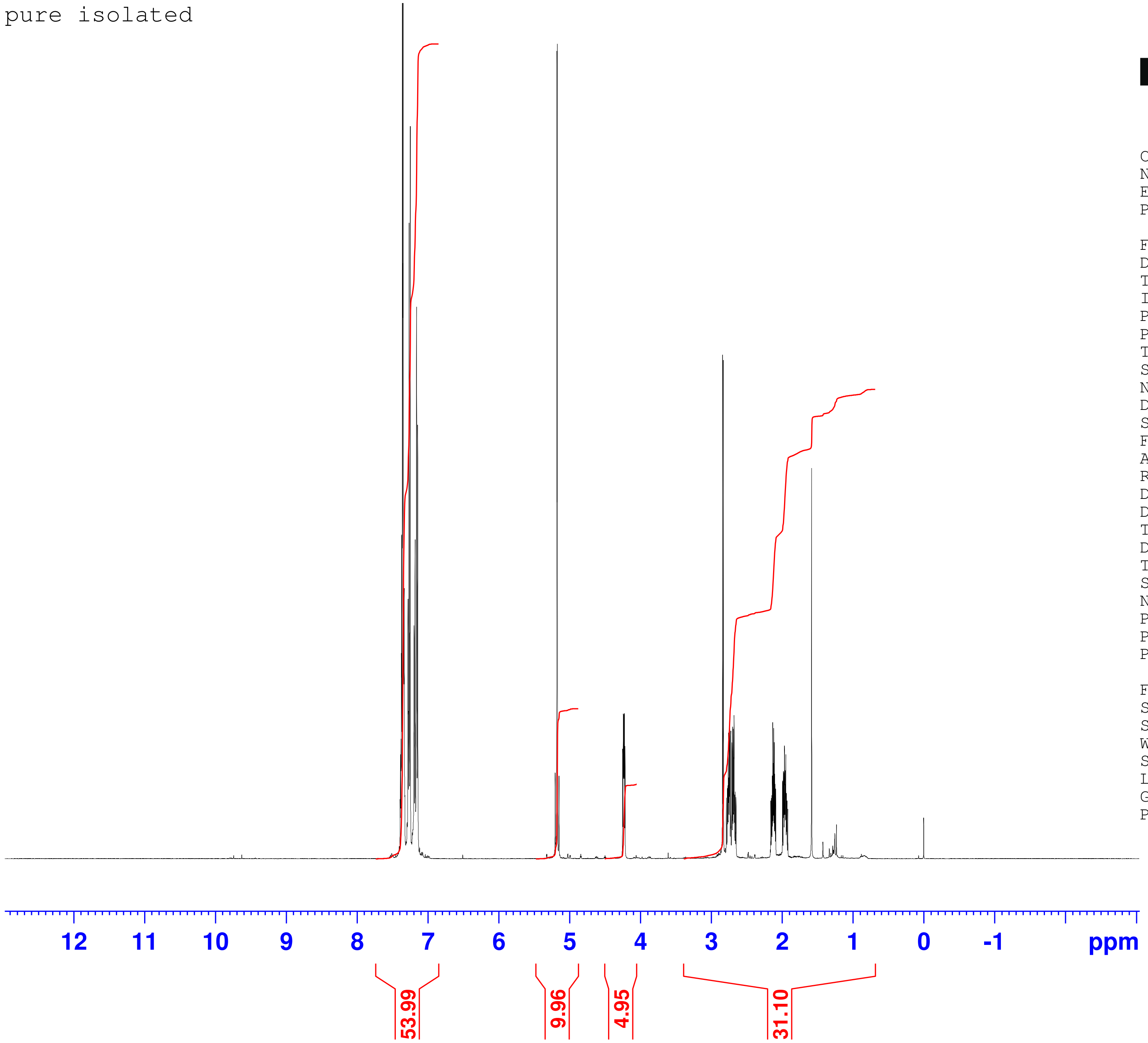

BRUKER

Current Data Parameters

NAME	VO_LA_S18
EXPNO	6
PROCNO	1

F2 - Acquisition Parameters

Date_	20250722
Time	13.21 h
INSTRUM	spect
PROBHD	Z114229_0012 (
PULPROG	zg30
TD	32768
SOLVENT	CDCl3
NS	8
DS	4
SWH	8012.820 Hz
FIDRES	0.489064 Hz
AQ	2.0447233 sec
RG	126.16
DW	62.400 usec
DE	17.43 usec
TE	298.0 K
D1	10.00000000 sec
TD0	1
SFO1	500.1325007 MHz
NUC1	1H
P0	4.00 usec
P1	12.00 usec
PLW1	18.50000000 W

F2 - Processing parameters

SI	65536
SF	500.1300173 MHz
WDW	EM
SSB	0
LB	0.30 Hz
GB	0
PC	1.00
