# Supplementary figures and images for: Synthesis of aliphatic α-hydroxy carboxylic acids via electrocarboxylation of aldehydes
Source: RSC Adv. 2025 Nov 21;15(53):45724–8. doi: 10.1039/d5ra07885g (PMC12637178; doi:10.1039/d5ra07885g)

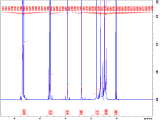

Supplement: RA-015-D5RA07885G-s001 [file RA-015-D5RA07885G-s001.zip › NMRSpectra_Isolated_products/Benzyl 2-hydroxy-3,3-dimethylbutanoate/1/pdata/1/thumb.png]
