# Supplementary material for: Synthesis of aliphatic α-hydroxy carboxylic acids via electrocarboxylation of aldehydes
Source: RSC Adv. 2025 Nov 21;15(53):45724–8. doi: 10.1039/d5ra07885g (PMC12637178; doi:10.1039/d5ra07885g)
Supplement: RA-015-D5RA07885G-s002 [file RA-015-D5RA07885G-s002.pdf]

Supplementary Information (SI) for RSC Advances.
This journal is © The Royal Society of Chemistry 2025

Table of Contents

Contents

1. Experimental 2
 1.1. General Information 2
 1.2. General Procedure for the Electrocarboxylation of Aldehydes 3
2. NMR Analysis 3
3. Screening and Optimisation Studies 5
 3.1. Screening of Cathodes 5
 3.2. Screening of Solvents and Anodes 6
 3.3. Screening of Counter Reactions for Anodic Oxidation 7
 3.4. Optimisation of Continuous Parameters 8
 3.5. Control Studies 10
4. Cyclic Voltammetry 15
5. Radical Trapping Experiment 18
6. Synthesis of Anhydrous Formaldehyde 19
7. Scope Studies 20
 7.1. Electrocarboxylation of Aliphatic Aldehydes 20
 7.2. Isolation of aliphatic hydroxy acids as benzyl esters 20
8. NMR spectra 24
9. Supporting Information References 30

1. Experimental

1.1. General Information

Chemicals: All chemicals and solvents were purchased and used without any purification unless otherwise stated. Acetaldehyde (>99%) was purchased from VWR, DMF was supplied by VWR (≥99.5%, max 0.1% water) or Sigma-Aldrich (≥99.0%, max 0.1% water) and used without drying unless otherwise specified. Carbon dioxide (99.8%) was supplied by Woikoski.

Electrodes: Graphite electrodes (C_{gr}, Sigrafine V2100) were purchased from SGL Carbon. Graphite surfaces were cleaned after reactions with a synthetic polyamide fiber polishing pad, after which they were sonicated for around ten minutes in a water bath, further washed with water and acetone, and finally polished with a paper towel and acetone. The glassy carbon (C_{GC}, Sigradur G, HTW Hochtemperatur-Werkstoffe GmbH) surface was washed with acetone and water and wiped with a paper towel prior to use. Boron-doped diamond electrodes (BDD, Merck) were conditioned by electrolyzing in 20% H_2SO_4 with BDD as an anode and C_{Gr}-plate as a cathode (10 C/cm², 10 mA/cm²) with subsequent rinsing with water and acetone.[1] Graphite felt electrodes ($C_{Gr\text{-}felt}$, SynLectro, Merck) were rinsed with acetone and air dried before use in electrolysis. Metal electrodes were purchased from Goodfellow (nickel and stainless steel (grade AISI 316)) or from Keskipakovalu Oy (CuSn7Pb15 and CuSn10Bi3) and the electrodes were polished with synthetic polyamide fiber polishing pad, after which they were rinsed with water and acetone, and polished with paper towel wetted with acetone. Electrode dimensions were 2 x 6 cm. During the electrolysis, the electrodes were submerged into solvent for 3 cm depth, leading to active surface area of 6 cm^2 and the interelectrode gap of 0.5 cm.

Electrolysis: The batch electrolysis experiments were performed in custom-manufactured undivided 25 mL glass cells with the diameter and height of the reactor part being 3 and 7 cm, respectively. The cell is a downscaled version of a commercially available SynLectro (Merck) glass cell developed by Waldvogel research group. CO_2 was continuously bubbled into electrolyte solution through a hole in the electrode holder. Electrode holders and PTFE stoppers were obtained from Merck. Electrolysis was powered with a four-channel power supply (Rohde & Schwarz HMP4040).

Analysis: NMR spectroscopy was used to monitor the conversion, yield, and selectivity of the reactions. All the spectra were acquired with Bruker Avance III 500 MHz Ultrashield spectrometer in $CDCl_3$ or DMSO-d_6 and referenced to tetramethylsilane (TMS, 0.00 ppm) or solvent residual peak (2.50 ppm, DMSO-d_6), respectively. All the isolated compounds were characterized with ^{1}H and ^{13}C NMR in $CDCl_3$ and compared to literature spectra. High-resolution mass spectra were obtained using Agilent 1260 Infinity High Performance Liquid

Chromatography (HPLC) System (Agilent Technologies, Singapore), coupled to a quadrupole time-of-flight mass spectrometer (6530 Accurate-Mass Q-TOF Agilent Technologies, Santa Clara, USA).

1.2. General Procedure for the Electrocarboxylation of Aldehydes

All the electroreduction experiments were conducted using a general procedure. First, tetraalkylammonium salt and possible additives were dissolved into 20 mL solvent(s) of choice in 25 mL undivided electrolysis cell under magnetic stirring (300 rpm). Next, the cell was closed by attaching the PTFE holder with the chosen electrodes. The solution was then presaturated by bubbling CO_2 into solution for 15 minutes and the bubbling was continued throughout the whole electrolysis time. Reactions were conducted by passing 2F charge of electrons through the electrodes unless otherwise stated. After the reaction, the electrode holder was removed. If the solution contained solid precipitates, the mixture was acidified with 1M HCl until a clear solution was obtained. Finally, 1,3,5-trimethoxybenzene (TMB) was added to the solution as an internal standard and the yields of the reaction products were analyzed with ^{1}H NMR.

2. NMR Analysis

Yields of all the products (lactic acid, ethanol and 2,3-butanediol) were recorded from the same sample, prepared by dissolving a few drops of reaction mixture that contained TMB into $CDCl_3$ or DMSO-d_6 (Figure S1 and S2). ^{1}H NMR spectra were acquired by accumulating 8 or 16 transients with a relaxation delay of 35s with zg30 pulse sequence. The standard processing of the spectra included phase and baseline correction (Whittaker Smoother) and the yields were calculated from the integrated spectra (Figures S1 and S2). Internal standard TMB (6.08 ppm, s, 3H) was integrated from 6.11 to 6.05 ppm. Lactic acid (4.05 ppm (in DMSO-d_6) or 4.28 ppm (in $CDCl_3$), q, 1H) were integrated from 4.08 to 4.01 ppm in DMSO-d_6 or from 4.31 to 4.24 ppm in $CDCl_3$. Ethanol (1.06 ppm (in DMSO-d_6) or 1.23 ppm (in $CDCl_3$), t, 3H) was integrated from 1.09 to 1.045 ppm in DMSO-d_6 or from 1.25 to 1.205 ppm in $CDCl_3$. 2,3-Butanediol appears as a mixture between *meso* and racemic forms and the yields are calculated from the sum of the two forms. 2,3-Butanediol peaks was integrated from 1.035 to 1.01 ppm and from 0.99 to 0.965 ppm in DMSO-d_6 (m, 6H) or from 1.175 to 1.125 ppm in $CDCl_3$ (m, 6H). Conversion of acetaldehyde (9.66 ppm (in DMSO-d_6) or 9.80 ppm (in $CDCl_3$), q, 1H) was characterized by integrating from 9.685 to 9.64 ppm in DMSO-d_6 or from 9.82 to 9.77 ppm in $CDCl_3$. Finally, molar amounts of each analyte were calculated and converted into yield and conversion.

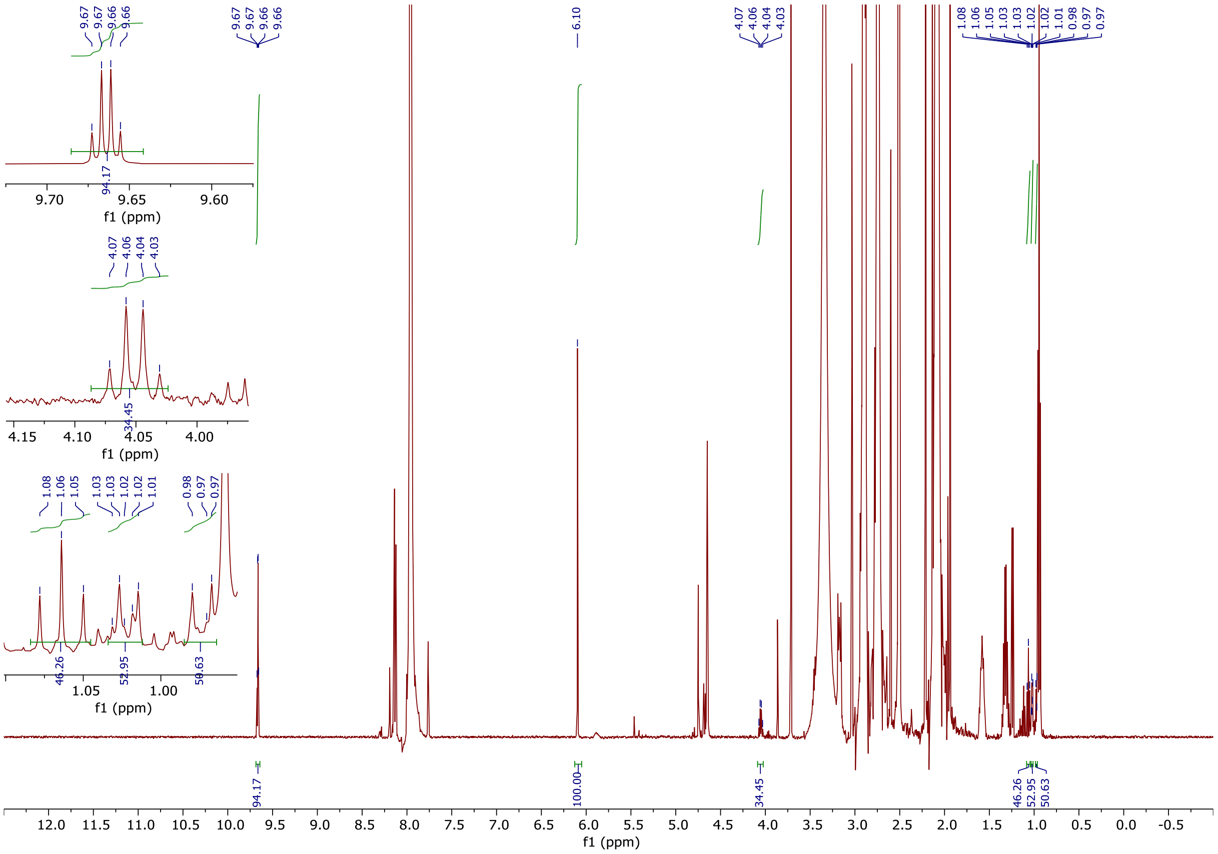

Figure S1: Representative example of NMR quantification of reaction products in DMSO-d$_6$, showing chemical shifts of acetaldehyde (9.66 ppm, q, 1H), TMB (6.10 ppm, s, 3H), lactic acid (4.05 ppm, q, 1H), ethanol (1.06 ppm, t, 3H) and 2,3-butanediol (1.03 – 1.01 ppm and 0.98 – 0.97 ppm, m, 6H)

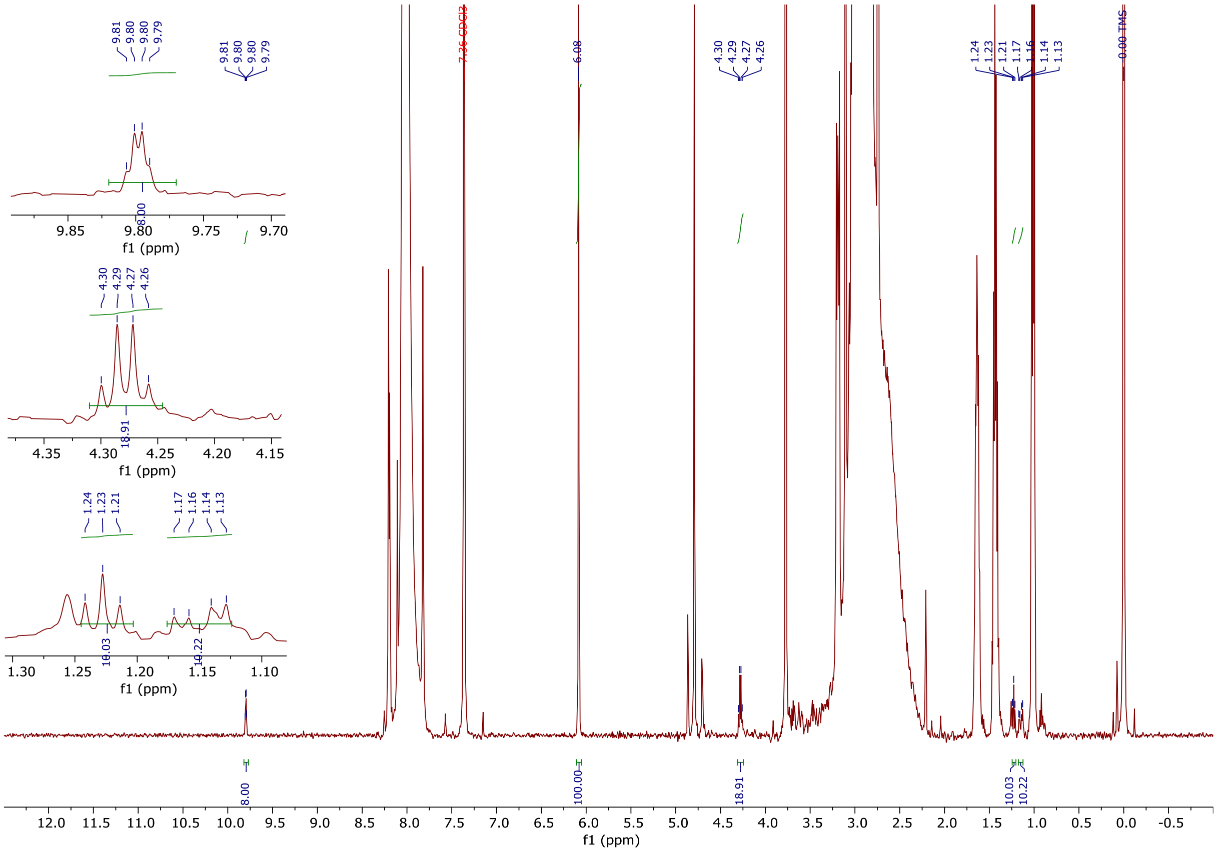

Figure S2: Representative example of NMR quantification of reaction products in $CDCl_3$ showing chemical shifts of acetaldehyde (9.80 ppm, q, 1H), TMB (6.08 ppm, s, 3H), lactic acid (4.28 ppm, q, 1H), ethanol (1.23 ppm, t, 3H) and 2,3-butanediol (1.17 – 1.13 ppm, m, 6H)

3. Screening and Optimisation Studies

3.1 Screening of Cathodes

We initiated our study by screening cathode materials with sacrificial aluminum anode (Al). The screening was performed according to the protocol described in section *1.2. General procedure for the electrocarboxylation of aldehydes*. All the reactions were performed using 2 mmol acetaldehyde (0.113 mL), dissolved in 20 mL DMF with 0.02 M tetrabutylammonium bromide as the supporting electrolyte. All the experiments were performed at room temperature with current density of 15 mA cm^{-2} until 2F charge was passed through. After the reaction, the solution was acidified with 1M HCl until the solution became clear. Results of the cathode screenings are summarized in Table S1.

Table S1. The effect of cathode material on the product distribution of aldehyde electroreduction in the presence of carbon dioxide.

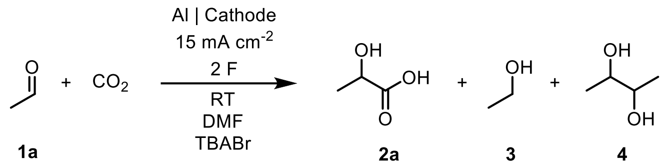

Entry	Cathode	1a Conversion (%)[a]	Yields (%)[a] 2a	3	4
1	Glassy carbon	96	7	4	5
2	Graphite	93	5	23	10
3	CuSn7Pb15-alloy	85	1	2	1
4	Nickel	100	2	2	4
5	RVC	99	5	8	14
6	Stainless Steel (AISI 316)	100	1	1	1
7	Boron-doped diamond	95	9	5	10
8	CuSn10Bi3-alloy	100	2	2	1
9	Graphite felt	100	5	15	8

[a] Yields and conversion determined by ^{1}H NMR using 1,3,5-trimethoxybenzene (TMB) as an internal standard

3.2. Screening of Solvents and Anodes

We also screened various solvents for electrocarboxylation of aldehydes. All the solvent screenings were performed according to section *1.2. General procedure for the electrocarboxylation of aldehydes* with 20 mL solvent and the results are summarized in Table S2. Supporting electrolyte was either tetrabutylammonium bromide (TBABr), tetrabutylammonium tetrafluoroborate ($TBABF_4$) and/or tetrabutylammonium hexafluorophosphate ($TBAPF_6$). Concentration of supporting electrolytes were 0.025 M except in Entries 2 and 3, where concentration of TBABr and $TBAPF_6$ were 0.02 M each. All the experiments were performed at room temperature with 2 mmol acetaldehyde, using a glassy carbon cathode with a current density of 15 mA cm^{-2} until 2F charge was passed through. The anode was either aluminum, glassy carbon or graphite. Entries with sacrificial Al anode were acidified with 1M HCl after electrolysis until clear solution was obtained.

Table S2. The effect of the solvent and anodes on the product distribution of aldehyde electroreduction in the presence of carbon dioxide.

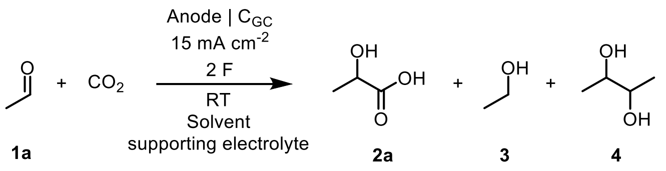

Entry	Solvent	Supporting electrolyte	Anode	1a Conversion (%)[a]	Yields (%)[a] 2a	3	4
1	N-Butylpyrrolidone (NBP)	TBABr	Al	54	0	0	0
2	MeCN	1:1 TBABr:$TBAPF_6$	Al	89	3	8	0
3	Propylene carbonate (PC)	1:1 TBABr:$TBAPF_6$	Al	89	trace	14	20
3	1:1 MeCN:DMF	$TBABF_4$	Gr	94	19	8	12
4	9:1 MeCN:DMF	$TBABF_4$	Gr	not recorded	14	5	14
5	MeCN	$TBABF_4$	Gr	67	0	5	0
6	1:1 MeCN:DMF	$TBABF_4$	GC	73	21	9	21

[a] Yields and conversion determined by ^1H NMR using 1,3,5-trimethoxybenzene (TMB) as an internal standard

3.3. Screening of Counter Reactions for Anodic Oxidation

The initial solvent screening indicated that DMF is essential for achieving electrocarboxylation of acetaldehyde in an undivided cell. When DMF is used as a co-solvent, most plausible counter reaction is DMF oxidation. Therefore, we wanted to investigate if other oxidation reactions would function as the anodic counter reactions using electrolyte consisting of 20 mL MeCN and/or DMF and 0.025 M $TBABF_4$ or $TBAPF_6$ with current density of 5 or 15 mA cm^{-2} (Table S3). For entries with ethanol as an additive, yield of ethanol from aldehyde reduction could not be recorded (Table S3, Entries 1 and 3). Similarly, 2,3-butanediol overlaps with triethylamine and thus its yield could not be recorded for Entry 2.

Table S3. The effect of the additives on acetaldehyde electrocarboxylation.

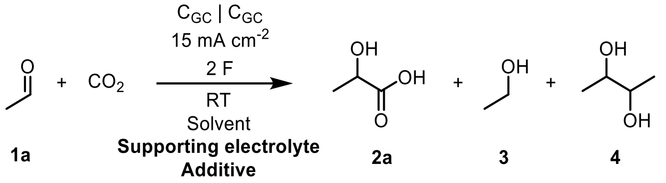

Entry	Solvent	Supporting electrolyte	Additive(s)	Current density (mA cm^{-2})	1a Conversion (%)[a]	Yields (%)[a] 2a	3	4
1	1:1 MeCN:DMF	$TBABF_4$	EtOH (1.7 equiv.) NHPI (0.3 equiv.)	15	98	2	-	2
2	1:1 MeCN:DMF	$TBABF_4$	Triethylamine (3.9 equiv.)	15	100	3	11	-
3	MeCN	$TBAPF_6$	EtOH (1 equiv.) TEMPO (0.25 equiv.)	5	75	10	-	0
4	1:1 MeCN:DMF	$TBABF_4$	KHCOO (1 equiv.)	5	88	0	5	4
5	DMF	$TBAPF_6$	TBD (1.2 equiv.)	5	74	3	3	3
6	MeCN	TBAI	TBAI (1.1 equiv.)	5	86	11	4	4
7	DMF	$TBAPF_6$	HCOOH (1 equiv.)	5	100	18	14	46

[a] Yields and conversion determined by ^{1}H NMR using 1,3,5-trimethoxybenzene (TMB) as an internal standard

3.4. Optimisation of Continuous Parameters

Two continuous parameters; temperature and current density were both optimized one at the time based on the procedure described in section *1.2. General procedure for the electrocarboxylation of aldehydes*. First, the reaction was conducted at three different temperatures in 1:1 MeCN:DMF mixture with (0.025 M) $TBABF_4$ as the supporting electrolyte to compare the effect of the temperature (Table 3, Entries 1 – 3). The highest lactic acid yield was obtained at room temperature (21%, Entry 2). At lowered 0 °C temperature (Entry 1), the yield of lactic acid was 19%, while both ethanol (11%) and 2,3-butanediol (29%) yields increased. On the contrary, a higher temperature of 40 °C (Entry 3) reduced yields of side products ethanol (8%) and butanediol (15%). However, yield of lactic acid was also reduced to 18%.

Table S4. The effect of temperature on electroreduction yields of acetaldehyde

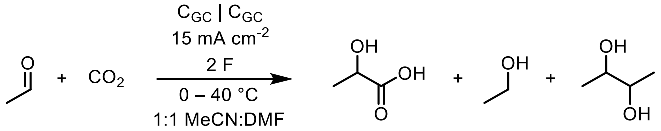

1a **2a** **3** **4**

Entry	Temperature (°C)	1a Conversion (%)[a]	Yields (%)[a] 2a	3	4
1	0	62	19	11	29
2	22	73	21	9	21
3	40	85	18	8	15

[a] Yields and conversion determined by 1H NMR using 1,3,5-trimethoxybenzene (TMB) as an internal standard

We then proceeded to optimize the current density for acetaldehyde reduction at room temperature, using either 1:1 MeCN:DMF or DMF as the solvent with 0.025 M $TBABF_4$ and $TBAPF_6$ supporting electrolytes (Table S5). In general, higher current densities gave higher combined selectivity for the three electroreduction products, but lower selectivity for the lactic acid. On the contrary, a higher lactic acid selectivity was obtained with low current densities in DMF. The best results were achieved using 5 mA cm^{-2} current density, which furnished lactic acid in 27 and 28% yields using either C_{GC} or BDD cathodes, respectively (Table S5, Entries 5 and 6). Further decrease of current density to 2.5 mA cm^{-2} reduced the yield of lactic acid to 10% (Entry 7), which might be due to increased acetaldehyde evaporation and conversion due to the increased reaction time.

Table S5. The effect of current density on the electroreduction yields of acetaldehyde

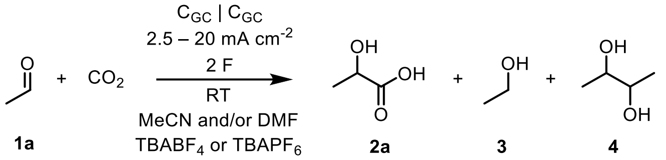

Entry	Current density (mA cm^{-2})	n (Acetaldehyde) (mmol)	Supporting electrolyte	Solvent	1a Conversion (%)[a]	Yields (%)[a]		
						2a	3	4
1	20	2	$TBABF_4$	1:1 MeCN:DMF	52	17	18	17
2	15	2	$TBABF_4$	1:1 MeCN:DMF	73	21	9	21
3	5	0.5	$TBABF_4$	1:1 MeCN:DMF	89	20	7	6
4	5	0.666	$TBAPF_6$	1:1 MeCN:DMF	78	20	7	7
5	5	1	$TBAPF_6$	DMF	88	27	4	4
6[b]	5	1	$TBAPF_6$	DMF	98	28	6	9
7	2.5	1	$TBAPF_6$	DMF	100	10	6	9
8[b]	10	2	$TBAPF_6$	DMF	72	22	12	20

[a] Yields and conversion determined by ^{1}H NMR using 1,3,5-trimethoxybenzene (TMB) as an internal standard. [b] BDD cathode instead of C_{GC}.

3.5. Control Studies

Control studies were performed according to the experimental set-up described in section *1.2. General procedure for the electrocarboxylation of aldehydes* with optimal reaction conditions as the baseline in comparison to the control studies (Table S6, Entry 1). All the control studies were performed using BDD cathode, C_{GC} anode at room temperature and DMF solution containing 0.025 M $TBAPF_6$ with acetaldehyde concentration of 0.05 M. The variation from the optimal conditions is expressed in Table S6. For the control experiments investigating the effect of water (Entries 6 – 9), DMF was dried by storing the solvent over dried 4 Å molecular sieves, which were activated in muffle-furnace at 400 °C. The effect of water was investigated

by using dry DMF (20 mL) with the addition of controlled amount of water, resulting in an electrolyte with varied water concentrations between 0.08 to 0.64 vol-% (Entries 7 – 9, Figure S3).

Table S6. Control studies on acetaldehyde reduction in the presence of CO_2

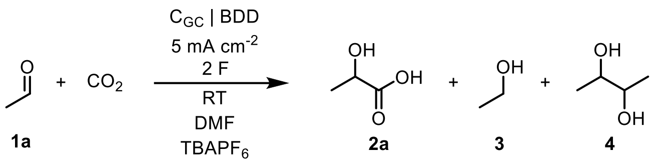

Entry	Variation from optimal conditions	1a Conversion (%)[a]	Yields (%)[a]		
			2a	3	4
1	none	97	28	6	10
2	no electricity	48	0	0	0
3	no continuous CO_2 bubbling, only presaturation	68	14	4	7
4[b]	0.2 F instead of 2 F	-	36	4	7
5[b]	0.5M acetaldehyde instead of 0.05M and 0.2 F instead of 2 F	-	9	17	60
6	DMF dried over 4Å molecular sieves	100	5	3	7
7	DMF with 0.08 vol-% water	98	25	7	12
8	DMF with 0.32 vol-% water	86	27	7	14
9	DMF with 0.64 vol-% water	91	30	10	18

[a]Yields determined by ^{1}H NMR using 1,3,5-trimethoxybenzene (TMB) as an internal standard. [b] Current efficiencies instead of yields.

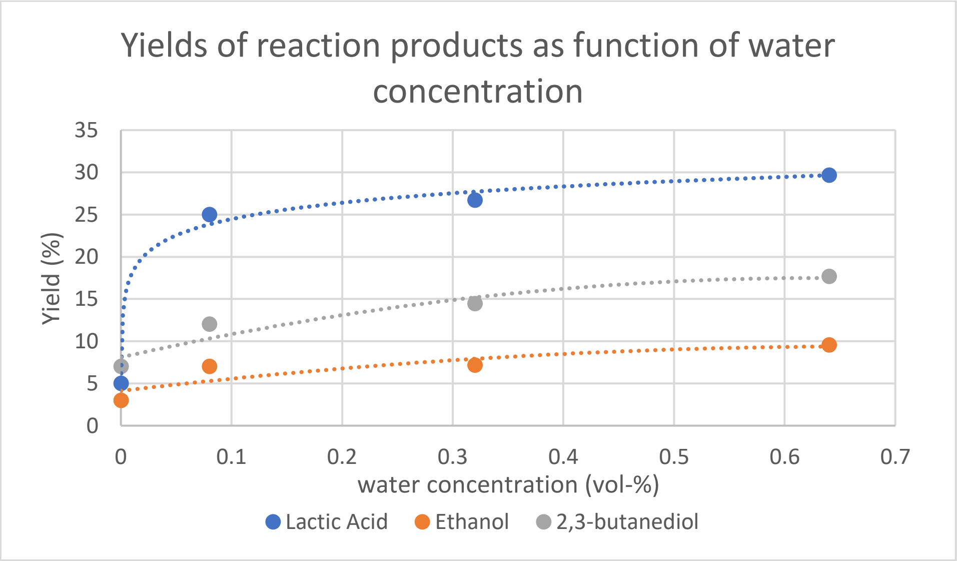

Figure S3. Yields of the reaction products as a function water concentration in the employed electrolyte.

3.6. Kinetic Study

Kinetic study was performed according to the general experimental set-up described in section *1.2. General procedure for the electrocarboxylation of aldehydes* with the optimized reaction conditions (Table S6, Entry 1). The conversion and product yields were monitored with ^{1}H NMR by stopping the electrolysis periodically and taking 0.050 mL sample for 0.2, 0.55, 0.9, 1.25, 1.6, 1.95 F charges. Then, samples were spiked with 0.025 mL 100 mg/mL 1,3,5-trimethoxybenzene, and diluted with $CDCl_3$. Shortly, the experiment (Table S7 and Figure S4) shows that the current efficiency remains practically constant between 35 and 38% for around two thirds of the electrolysis time (Table S7, Entries 1 – 4). Only after 1.25 F charge has been passed, the current efficiency for lactic acid decreases (Entries 5 and 6), yet despite the decrease in current efficiency, the amount of lactic acid is increased until electrolysis is stopped at 1.95 F (Entry 6).

Table S7. Kinetic experiment for electrocarboxylation of acetaldehyde

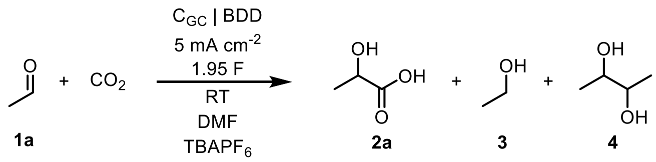

Entry	Charge (F)	1a Conversion (%)[a]	Yields (%)[a] 2a	3	4	Current Efficiency(%)[a] 2a	3	4
1	0.2	51	4	0	1	36	4	7
2	0.55	61	10	1	5	35	5	9
3	0.9	74	17	4	8	38	8	9
4	1.25	85	22	5	10	36	8	8
5	1.6	93	23	5	10	28	6	6
6	1.95	98	25	7	12	25	7	6

[a]Yields determined by ^{1}H NMR using 1,3,5-trimethoxybenzene (TMB) as an internal standard

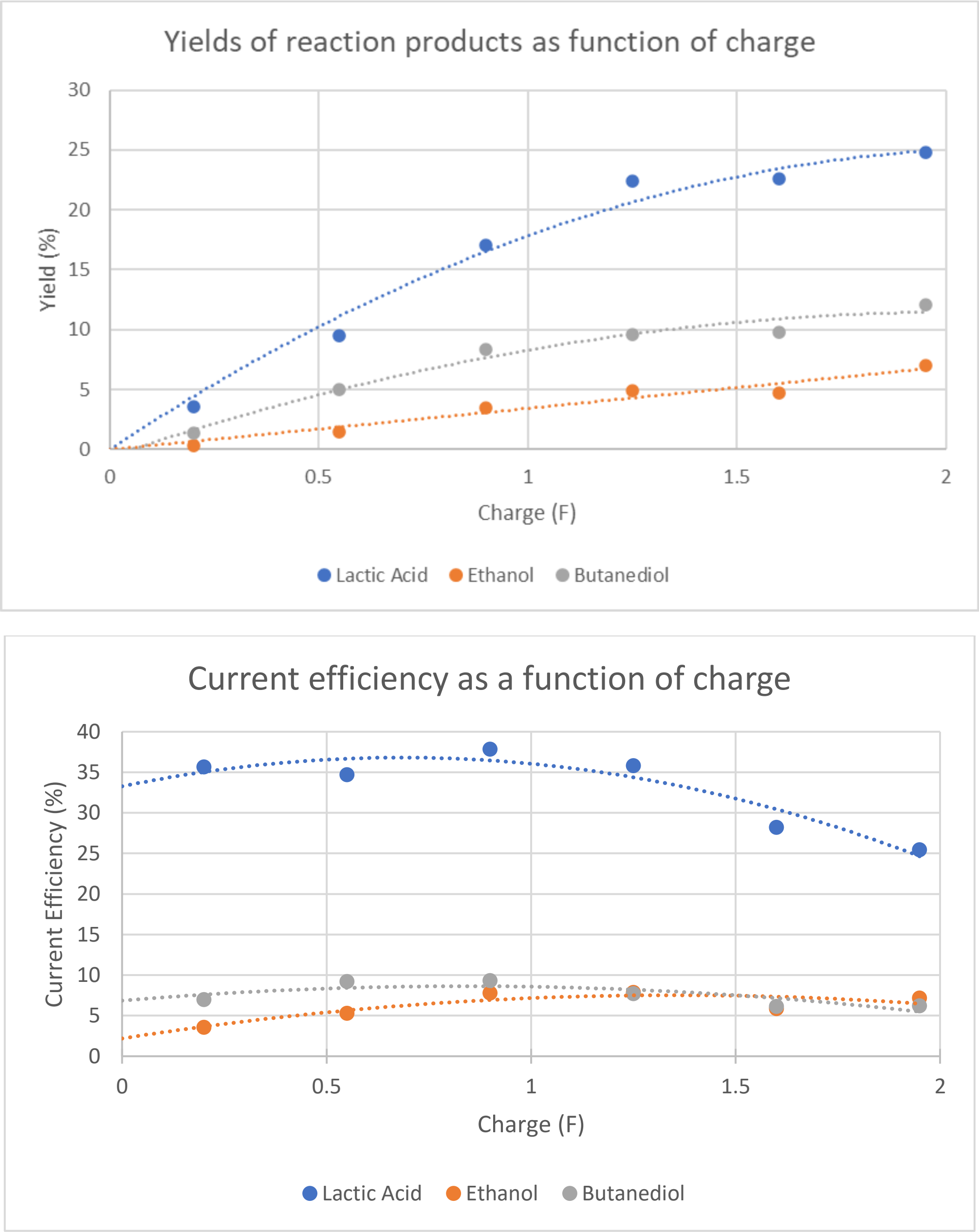

Figure S4. Yields (up) and current efficiencies (down) of the reaction products as a function of applied charge.

3.7. Inhibitor Tolerance

We investigated the tolerance of the electrocarboxylation to a range of functional groups that were present either as reaction solvent or additive. Based on the screening of counter reactions (Table S3), the presence of one equivalent formic acid (Table S3, Entry 7) did not inhibit the reaction and 18% lactic acid was obtained. In addition, amide (DMF) and nitrile (MeCN) solvents are well tolerated. Conversely, the presence of amine (triethylamine) or guanidine (triazabicyclodecene, TBD) leads to almost complete inhibition of the reaction (Tables S3, Entries 2 and 5), producing only 3% of lactic acid. In order to investigate robustness of the

developed electrocarboxylation protocol further, we studied the effect of various inhibitive additives for the electrocarboxylation. We used acetaldehyde **1a** (1 mmol) and nonanal **1e** (2 mmol) as the model substrates, using optimized reaction conditions with BDD or glassy carbon cathode (Table S8). Presence of zinc dichloride, led into complete inhibition of the reaction and electroplating of the employed glassy carbon electrode (Table S8, Entry 1). Furthermore, the presence of 0.5 mmol 1,4-butynediol completely inhibited the formation of the lactic acid (Entry 2) while the formation of 2-hydroxydecanoic acid **2e** from **1e** was completely inhibited in the presence of trimethylsilyl chloride (TMSCl) and acetic anhydride (Ac_2O) (Entries 6 and 8). Conversely, the presence of isopropanol (IPA) did not lead into diminishing of **2e** formation (Entry 7). In fact, the yield was improved to 28% in comparison to optimized reaction conditions, which yielded 22% (Main body, Scheme 2). Furthermore, the presence of 0.5 mmol chloro- and iodobenzene (PhCl and PhI) was tolerated although significantly diminish lactic acid yield of 13 and 7% was obtained, with PhI resulting in more profound decrease in the yields of other reduction products as well (Entries 2 and 3). In addition, hexafluoroisopropanol (HFIP) was tolerated, resulting in reduced **2e** formation with 13% yield.

Table S8. Effect of inhibitors for electrocarboxylation of acetaldehyde and nonanal

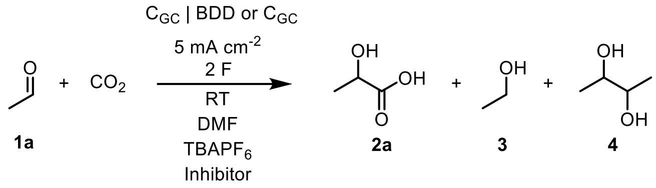

Entry	Cathode	Inhibitor	Yields (%)[a] 2a	3	4
1	GC	$ZnCl_2$ (1.1 mmol)	0	0	0
2	BDD	1,4-Butynediol (0.5 mmol)	0	1	2
3	BDD	PhCl (0.5 mmol)	13	3	8
4	BDD	PhI (0.5 mmol)	7	5	3

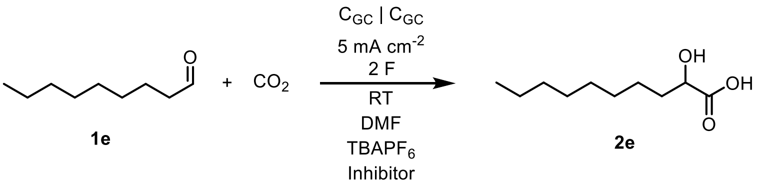

Entry	Cathode	Inhibitor	2e
5	GC	HFIP (2 mmol)	13
6	GC	TMSCl (2 mmol)	0
7	GC	IPA (2 mmol)	28
8	GC	Ac_2O (2 mmol)	0

[a]Yields determined by ^1H NMR using 1,3,5-trimethoxybenzene (TMB) as an internal standard

4. Cyclic Voltammetry

Voltammetric curves for acetaldehyde reduction in Ar and CO_2 -saturated solutions and for oxidative counter reactions were recorded with OrigaFlex - OGF05A Potentiostat (Origalys) connected to a power supply module (OGFPWR, Origalys). All voltametric studies were conducted in a flame-dried 50 mL electrochemical glass cell (BEC 50 ML, organic electrolyte, Redox.me). The cell was equipped with circular disk working electrode (glassy carbon, 2mm diameter, Redox.me), custom-made non-aqueous Ag/Ag^+ refillable reference electrode (filled with solution of 0.01 M $AgNO_3$ and 0.1 M $TBAPF_6$ in DMF, Redox.me) and platinum wire auxiliary electrode (Redox.me). Prior to voltametric measurements, the working electrode was polished using a microcloth pad and alumina (0.05 µm Al_2O_3) slurry.

Reductive scans (Figure S5) were performed with a C_{GC} working electrode with a scan rate of 100 mV/s. Potential was scanned starting from 0.0 V and scan was continued to -2.5 V vs Ag/Ag^+ reference. Baseline of both Ar and CO_2 saturated was recorded after presaturating 0.1 M $TBAPF_6$ DMF electrolyte for 15 minutes with the respective gas. After measuring CV for presaturated DMF, acetaldehyde was added to the solution and CVs were recorded for acetaldehyde (0.044 M) in Ar and CO_2 saturated solutions. Finally, ferrocene (tip of the spatula) was added to the solution to record the potential against the Fc/Fc^+ couple.

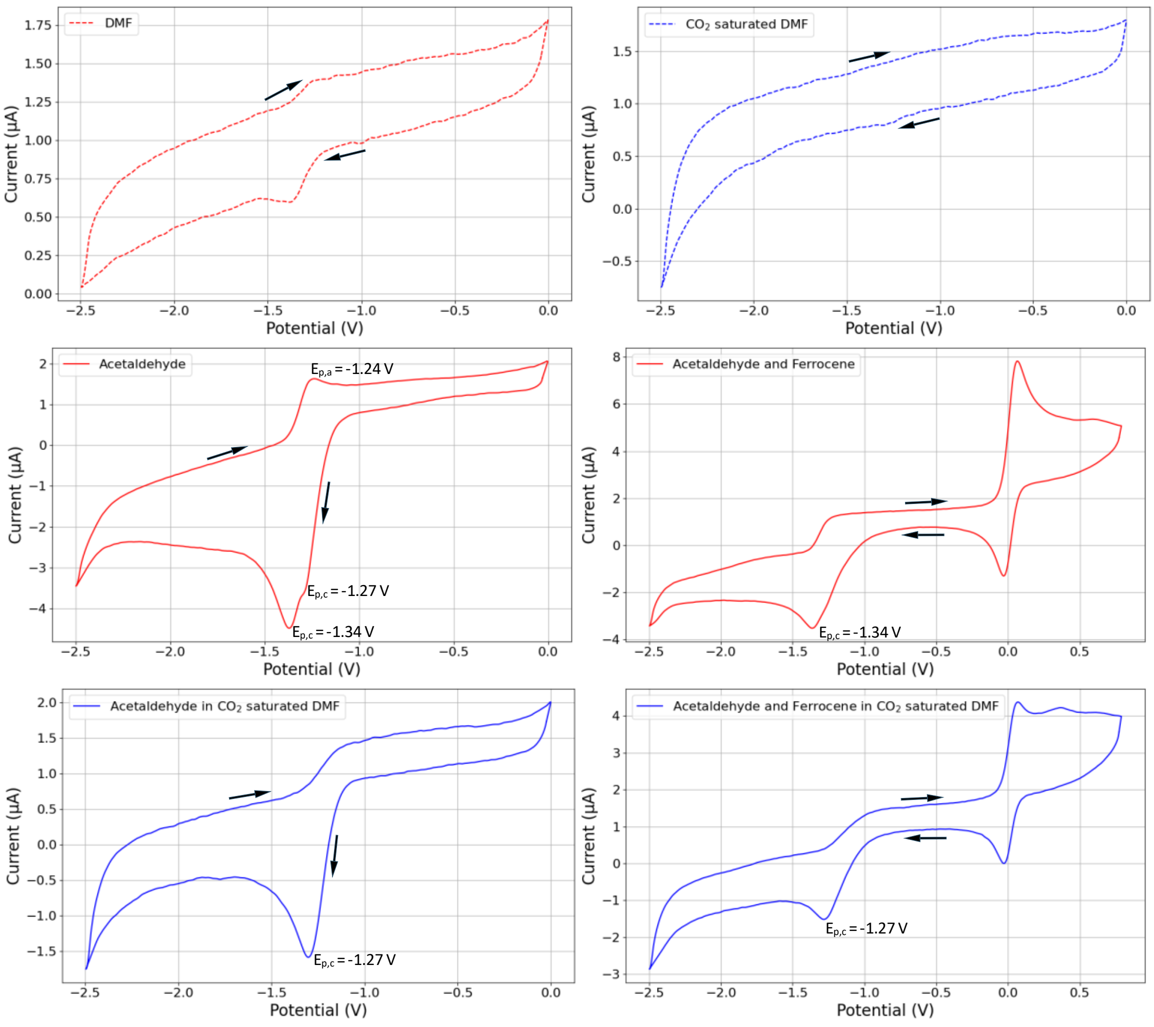

Figure S5. Cyclic voltammograms of solvent baselines and acetaldehyde (0.044M) in Ar (red) and CO_2 (blue) saturated 0.1M $TBAPF_6$ DMF solution with a scan rate of 100 mV/s. Potential was scanned from 0.0 or 0.8 V to -2.5 V vs. Ag/AgCl reference electrode. All the peak potentials in the figure are reported against Fc/Fc^+ redox couple.

Oxidative scans (Figure S6) were performed for DMF and possible reaction products by using the same set up as for the reductive scans: a C_{GC} working electrode, Ag/AgCl reference electrode and Pt-wire counter electrode. The scan rate was set to 100 mV/s. Potential was scanned from 0.0 V up to 1.05 V vs. Ag/AgCl electrode. Concentrations of ethanol, 2,3-butanediol and lactic acid were 0.033 M. Sodium lactate was prepared *in-situ* by adding 1 mmol sodium carbonate to 30 mL 0.033 M lactic acid solution in DMF. Noteworthy, sodium carbonate is only partly soluble in DMF. We then performed CV for the reaction mixture after the electrolysis, by adding $TBAPF_6$ to the reaction mixture until concentration relative to $TBAPF_6$ was 0.1 M. We then spiked the mixture with excess of sodium carbonate and compared the voltammogram to the one obtained from the reaction mixture. All the cyclic voltammograms were refenced against Fc/Fc^+ couple by adding a tip of the spatula of errocene into the solution.

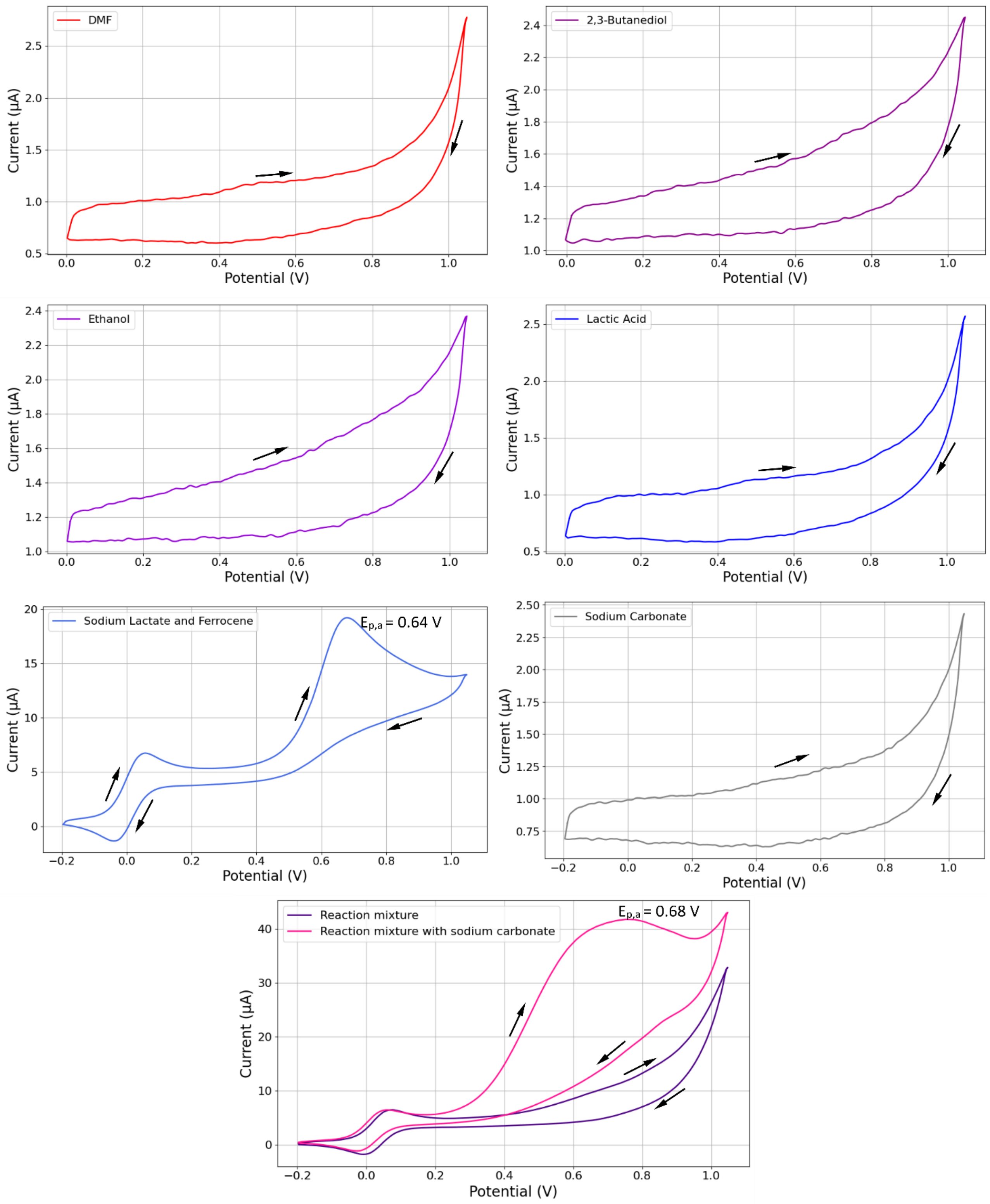

Figure S6. Cyclic voltammograms of DMF, various reaction products (0.033 M) in 0.1M $TBAPF_6$ DMF solution and reaction mixtures (0.014 M Lactic acid) with scan rate of 100 mV/s. Potential was scanned from -0.2 or 0.0 V to 1.05 V vs. Ag/AgCl reference electrode. All the peak potentials in the figure are reported against Fc/Fc^+ redox couple.

5. Radical Trapping Experiment

Sample for radical trapping experiment was collected by adding N-cyclohexyl-2-(((2,2,6,6-tetramethylpiperidin-1-yl)oxy)methyl)acrylamide (CHANT) to the reaction mixture before electrolysis. CHANT was prepared according to a literature procedure[2] and electrolysis was performed according to the general procedure 1.2. using BDD cathode, C_{GC} anode, and 1 mmol acetaldehyde that was electrolyzed for 2 F charge with current density of 5 mA cm^{-2}.

After the reaction, reaction mixture with added CHANT was analyzed by Agilent 1260 Infinity High Performance Liquid Chromatography (HPLC) System (Agilent Technologies, Singapore), coupled to a quadrupole time-of-flight mass spectrometer (6530 Accurate-Mass Q-TOF Agilent Technologies, Santa Clara, USA). Mass accuracy of the instrument using external calibration is specified to be ≤ 3ppm. The ionisation was done using Dual ESI in the positive ion mode. Analysis was performed by injecting 10 µL sample on a ZORBAX SB-C18 column (Solvent Saver Plus 3.0 × 75 mm, 3.5 Micron, Agilent, USA). A separation gradient of two mobile phases: 0.1% formic acid in water (A) and 0.1% formic acid in acetonitrile (B) was applied at a flow rate of 0.4 mL min–1. The gradient program is presented in the table below. The instrumental parameters were set as follows: capillary voltage 3500 V, gas temperature 300 °C with a flow of 12 L min-1, nebulizer pressure 25 psi, fragmentor 150 V, skimmer 65 V and octupole RF 500 V. Mass measurement was done with mass range m/z 100–1100.

Time (min)	A (%)	B (%)
5.00	80	20
20.00	50	50
30.00	50	50
45.00	20	80
65.00	20	80
70.00	5	95
80.00	5	95
85.00	80	20
100.00	80	20

HR-ESI-MS measurements from this mixture displayed a mass matching for the addition product **A,** which could form *via* combination of CHANT and formyloxyl radical **XII** (Scheme S1).

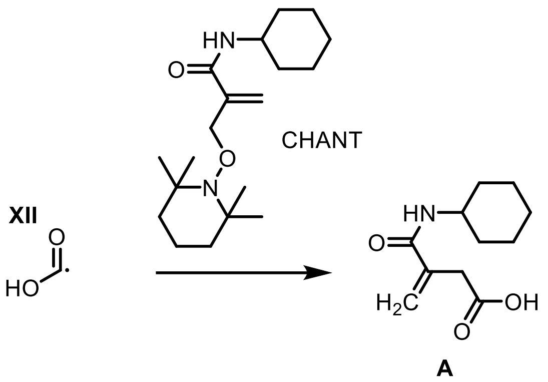

Scheme S1. Radical trapping experiment with CHANT

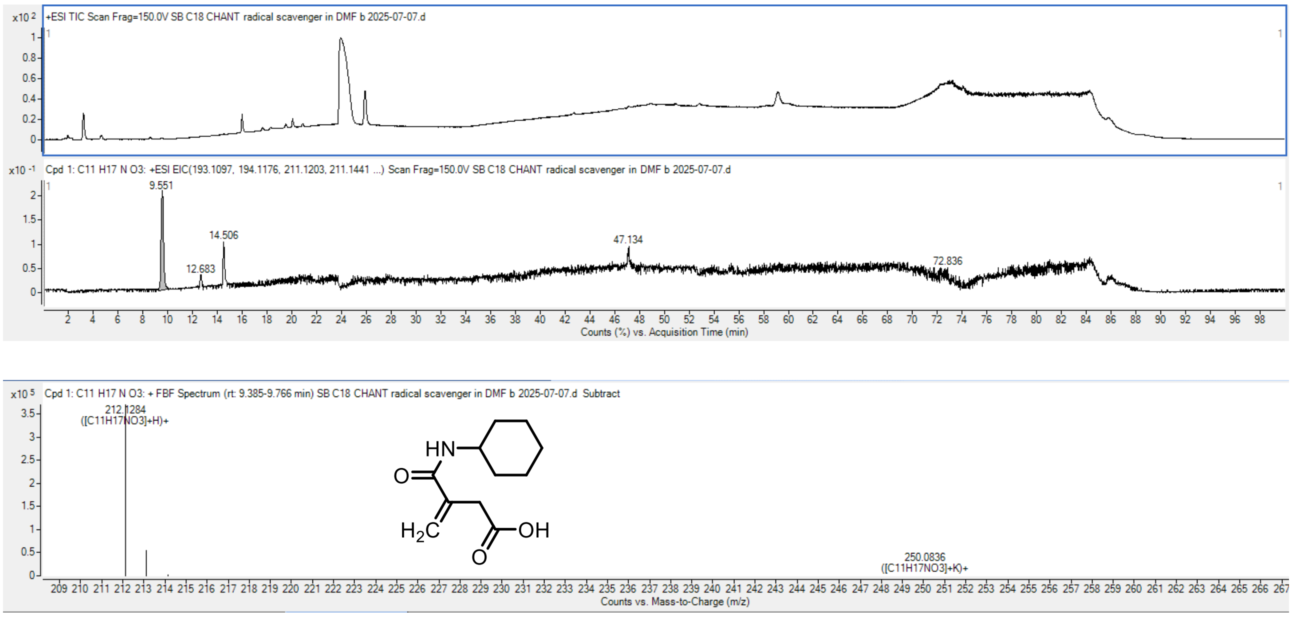

Figure S7. HPLC/HR-ESI-MS trace of adduct **A** from the reaction according to Scheme S1. Mass calculated for [C11H17NO3 + H]$^+$ 212.1287 found: 212.1284 (Δ = -1.41 ppm).

6. Synthesis of Anhydrous Formaldehyde

Anhydrous formaldehyde was prepared by depolymerization of paraformaldehyde. First, paraformaldehyde (96%) was weighed into a dried two-necked round-bottom flask, equipped with a magnetic stirrer. The flask was connected with a Teflon tubing into a nitrogen gas flow through one neck and into dried electrolyte solution through the other neck. The collection flask was equipped with a septum and needle to vent off the excess gas. The nitrogen gas flow was turned on and the flask with paraformaldehyde was then lowered into oil bath, preheated to 160 °C. The Teflon tubing was then lowered under the solvent level in the collection flask and nitrogen gas flow was used to carry gaseous formaldehyde into electrolyte in the collection flask. Paraformaldehyde was heated under stirring for a total of 10 minutes, after which the nitrogen gas flow was turned off and flask removed from the oil bath. The concentration of free formaldehyde in the electrolyte

solution was analysed with ^{1}H NMR[3] and the electrolyte solution was used instantly for the electrocarboxylation of the formaldehyde into a glycolic acid.

7. Scope Studies

7.1. Electrocarboxylation of Aliphatic Aldehydes

The scope of the electrocarboxylation reaction of aldehydes was studied with a range of aliphatic aldehydes. Anhydrous formaldehyde was obtained according to procedure described in Chapter 5, whereas the rest of the aldehydes were used as received from commercial suppliers. All the aldehyde carboxylations were carried out according to general procedure described in Section 1.2. using 5 mA cm^{-2} current density with C_{GC} anode and BDD cathode in 20 mL electrolyte consisting of 0.025 M $TBAPF_6$ solution in DMF presaturated with CO_2. Concentration of aldehydes were 0.5 M except for anhydrous formaldehyde, where two entries with 0.225 M and 0.0225 M formaldehyde concentrations were recorded. After electrolyzing the solution for 2F charge, 0.500 mL sample was taken from the solution while rest of the solution was saved for isolation of the product. The removed sample was spiked with 0.050 mL 100 mg/ml solution of 1,3,5-TMB in DMF. Part of this solution (0.100 mL) was diluted with 0.500 mL $CDCl_3$ and the yield of hydroxy acid products were calculated from integrals obtained from ^{1}H NMR spectra of the samples.

7.2. Isolation of aliphatic hydroxy acids as benzyl esters

The synthesized alpha hydroxy acids were isolated as corresponding benzyl esters due to the high hydrophilicity of some of the low molecular weight products. After the electrolysis and NMR sample preparation, the remaining solution was heated to 70 °C. To the preheated solution, triethylamine (1.2 mmol) was added, and the solution was mixed for 30 minutes. Then, benzyl bromide (1.2 mmol) was added into the solution which was mixed for further two hours at 70 °C. After the reaction was completed, DMF was removed in a vacuum and the residue was dissolved in a mixture of 20 mL DCM and 20 mL water. The organic phase was collected, and the water phase was extracted with 2 x 20 mL DCM. The combined organic phase was washed with 20 mL brine, dried with Na_2SO_4, and the solvent was evaporated in a vacuum. The resulting crude mixture was purified using a column chromatography (SiO_2). All the yields are reported relative to the aldehyde.

This protocol was also applied for esterification of pure lactic acid (1 mmol, 90.1 mg) in DMF, which provided benzyl lactate in 77% yield after evaporation of solvents.

Benzyl lactate 3a

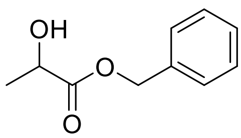

Benzyl lactate **3a** was synthesized via electrocarboxylation of acetaldehyde into lactic acid **2a** according to general procedure for electrocarboxylation described in section 7.1, followed by esterification with benzyl bromide into benzyl lactate **3a** according to general procedure for esterification, described in section 7.2. The crude was purified by a column chromatography (silica gel, 5/1 CyHex/EtOAc) affording benzyl lactate as a clear oil (21 mg, 12%). The NMR spectroscopic data is in agreement with the literature. [4]

^{1}H-NMR ($CDCl_3$, 500 MHz) δ: 7.45 – 7.28 (m, 5H), 5.21 (s, 2H), 4.32 (qd, *J* = 7.0, 5.3 Hz, 1H), 2.84 (d, *J* = 5.3 Hz, 1H), 1.43 (d, *J* = 7.0 Hz, 3H).

^{13}C-NMR ($CDCl_3$, 125 MHz) δ: 175.6, 135.2, 128.7, 128.6, 128.3, 67.3, 66.8, 20.4

Phenylmethyl 2-hydroxybutanoate 3c

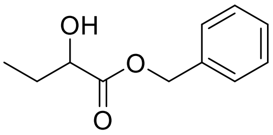

Phenylmethyl 2-hydroxybutanoate **3c** was synthesized via electrocarboxylation of propanal into 2-hydroxybutyric acid **2c** according to general procedure for electrocarboxylation described in section 7.1, followed by esterification with benzyl bromide into **3c** according to general procedure for esterification, described in section 7.2. The crude was purified by a column chromatography (silica gel, 8:1 CyHex/EtOAc) affording phenylmethyl 2-hydroxybutanoate as a clear oil (30 mg, 15%). The NMR spectroscopic data is in agreement with the literature.[5]

^{1}H-NMR ($CDCl_3$, 500 MHz) δ: 7.49 – 7.27 (m, 5H), 5.30 – 5.10 (m, 2H), 4.20 (ddd, *J* = 6.8, 5.7, 4.3 Hz, 1H), 2.77 (d, *J* = 5.7 Hz, 1H), 1.89 – 1.81 (m, 1H), 1.70 (dq, *J* = 14.2, 7.3 Hz, 1H), 0.94 (t, *J* = 7.5 Hz, 3H).

^{13}C-NMR ($CDCl_3$, 125 MHz) δ: 175.1, 135.2, 128.7, 128.6, 128.3, 71.5, 67.3, 27.5, 8.9

Benzyl 2-hydroxy-3,3-dimethylbutanoate 3d

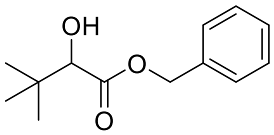

Benzyl 2-hydroxy-3,3-dimethylbutanoate **3d** was synthesized via electrocarboxylation of 2,2-dimethylpropanal into 2-hydroxy-3,3-dimethylbutanoic acid **2d** according to general procedure for electrocarboxylation described in section 7.1, followed by esterification with benzyl bromide into **3d** according to general procedure for esterification, described in section 7.2. The crude was purified by a column chromatography (silica gel, 16/1 CyHex/EtOAc) affording the product as a clear oil (26 mg, 12%). The NMR spectroscopic data is in agreement with the literature. [6]

^{1}H-NMR ($CDCl_3$, 500 MHz) δ: 7.40 – 7.33 (m, 5H), 5.25 – 5.18 (m, 2H), 3.85 (d, *J* = 7.7 Hz, 1H), 2.76 (d, *J* = 7.7 Hz, 1H), 0.95 (s, 9H).

^{13}C-NMR ($CDCl_3$, 125 MHz) δ: 174.4, 135.1, 128.7, 128.6, 128.5, 78.5, 67.3, 35.5, 25.8

Phenylmethyl 2-hydroxydecanoate 3e

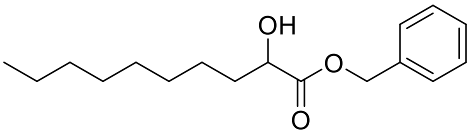

Phenylmethyl 2-hydroxydecanoate **3e** was synthesized via electrocarboxylation of nonanal into 2-hydroxydecanoic acid **2e** according to general procedure for electrocarboxylation described in section 7.1, followed by esterification with benzyl bromide into **3e** according to general procedure for esterification, described in section 7.2. The crude was purified by a column chromatography (silica gel, 16:1 CyHex/EtOAc) affording the product as a clear oil (24 mg, 9%).

^{1}H-NMR ($CDCl_3$, 500 MHz) δ: 7.46 – 7.28 (m, 5H), 5.28 – 5.16 (m, 2H), 4.22 (ddd, *J* = 7.3, 5.9, 4.2 Hz, 1H), 2.68 (d, *J* = 5.9 Hz, 1H), 1.84 – 1.75 (m, 1H), 1.69 – 1.60 (m, 1H), 1.45 – 1.38 (m, 1H), 1.38 – 1.16 (m, 11H), 0.87 (t, *J* = 7.0 Hz, 3H).

^{13}C-NMR ($CDCl_3$, 125 MHz) δ: 175.3, 135.2, 128.7, 128.6, 128.4, 70.5, 67.3, 34.4, 31.8, 29.4, 29.3, 29.2, 24.7, 22.7, 14.1

HRMS (ESI) calculated for $C_{17}H_{26}O_3$ $[M+Na^+]$: 301.1774. Found: 301.1781 (Δ = 2.32 ppm)

Phenylmethyl α-hydroxycyclohexaneacetate 3f

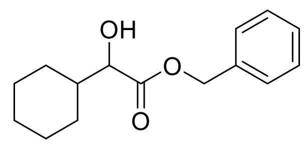

Phenylmethyl α-hydroxycyclohexaneacetate **3f** was synthesized via electrocarboxylation of cyclohexanecarboxaldehyde into 2-Cyclohexyl-2-hydroxyacetic acid **2f** according to general procedure for electrocarboxylation described in section 7.1, followed by esterification with benzyl bromide into **3f** according to general procedure for esterification, described in section 7.2. The crude was purified by a column chromatography (silica gel, 10:1 CyHex/EtOAc) affording the product as a clear oil (21 mg, 9%). The NMR spectroscopic data is in agreement with the literature. [7]

^{1}H-NMR ($CDCl_3$, 500 MHz) δ: 7.43 – 7.30 (m, 5H), 5.25 – 5.18 (m, 2H), 4.06 (dd, *J* = 6.4, 3.5 Hz, 1H), 2.67 (d, *J* = 6.4 Hz, 1H), 1.79 – 1.60 (m, 5H), 1.41 – 1.35 (m, 1H), 1.30 – 1.07 (m, 5H).

^{13}C-NMR ($CDCl_3$, 125 MHz) δ: 174.8, 135.3, 128.7, 128.6, 128.4, 74.9, 67.2, 42.0, 29.1, 26.3, 26.2, 26.0, 25.9

Phenylmethyl α-hydroxybenzenebutanoate 3g

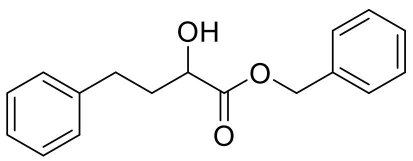

Phenylmethyl α-hydroxybenzenebutanoate **3g** was synthesized via electrocarboxylation of 3-phenylpropionaldehyde into 2-hydroxy-4-phenylbutyric acid **2g** according to general procedure for electrocarboxylation described in section 7.1, followed by esterification with benzyl bromide into **3g** according to general procedure for esterification, described in section 7.2. The crude was purified by a column chromatography (silica gel, 19:1 CyHex/EtOAc) affording the product as a clear oil (39 mg, 14%). The NMR spectroscopic data is in agreement with the literature. [8]

^{1}H-NMR ($CDCl_3$, 500 MHz) δ: 7.43 – 7.33 (m, 5H), 7.29 – 7.26 (m, 2H), 7.21 – 7.15 (m, 3H), 5.21 – 5.16 (m, 2H), 4.24 (ddd, J = 7.7, 5.5, 4.0 Hz, 1H), 2.85 (d, J = 5.5 Hz, 1H), 2.79 – 2.65 (m, 2H), 2.17 – 2.1 (m, 1H), 2.00 – 1.93 (m, 1H).

^{13}C-NMR ($CDCl_3$, 125 MHz) δ: 175.1, 141.1, 135.1, 128.7, 128.7, 128.6, 128.5, 128.4, 126.1, 69.8, 67.5, 36.0, 30.9

8. Comparison Between the Present and Previous Methods for Electrocarboxylation of Aliphatic Aldehydes

The present work and previous studies on the electrocarboxylation of aliphatic aldehydes were compared based on literature searchs. Only two works had previously described the electrosynthesis of aliphatic α-hydroxy acids[9,10] and comparison to yields obtained in these studies is presented in Table S9.

Table S9: Comparison between previous studies on the electrocarboxylation of aliphatic aldehydes and the present work

**Product**	**Conditions**	**AHA yield (%)**	**Reference**
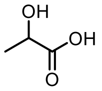	Al anode, Zn anode, DMF, undivided cell, 0.1 M TBABr, 5 bar CO_2	9	Silvestri *et al.*[9]
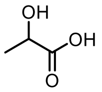	C_{GC} anode, BDD cathode, DMF, undivided cell, 0.025 M $TBAPF_6$, CO_2 bubbling, 5 mA cm^{-2}	28	**this work**
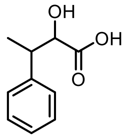	C_{Gr} anode and cathode, propylene carbonate, divided cell, 0.3 M TBAI, CO_2, 8 mA cm^{-2}	26	Vieira and Waldvogel *et al.*[10]
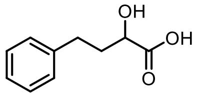	C_{Gr} anode and cathode, propylene carbonate, divided cell, 0.3 M TBAI, CO_2, 8 mA cm^{-2}	8	Vieira and Waldvogel *et al.*[10]
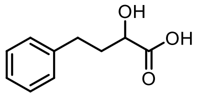	C_{GC} anode, BDD cathode, DMF, undivided cell, 0.025 M $TBAPF_6$, CO_2 bubbling, 5 mA cm^{-2}	26	**this work**

9. NMR spectra

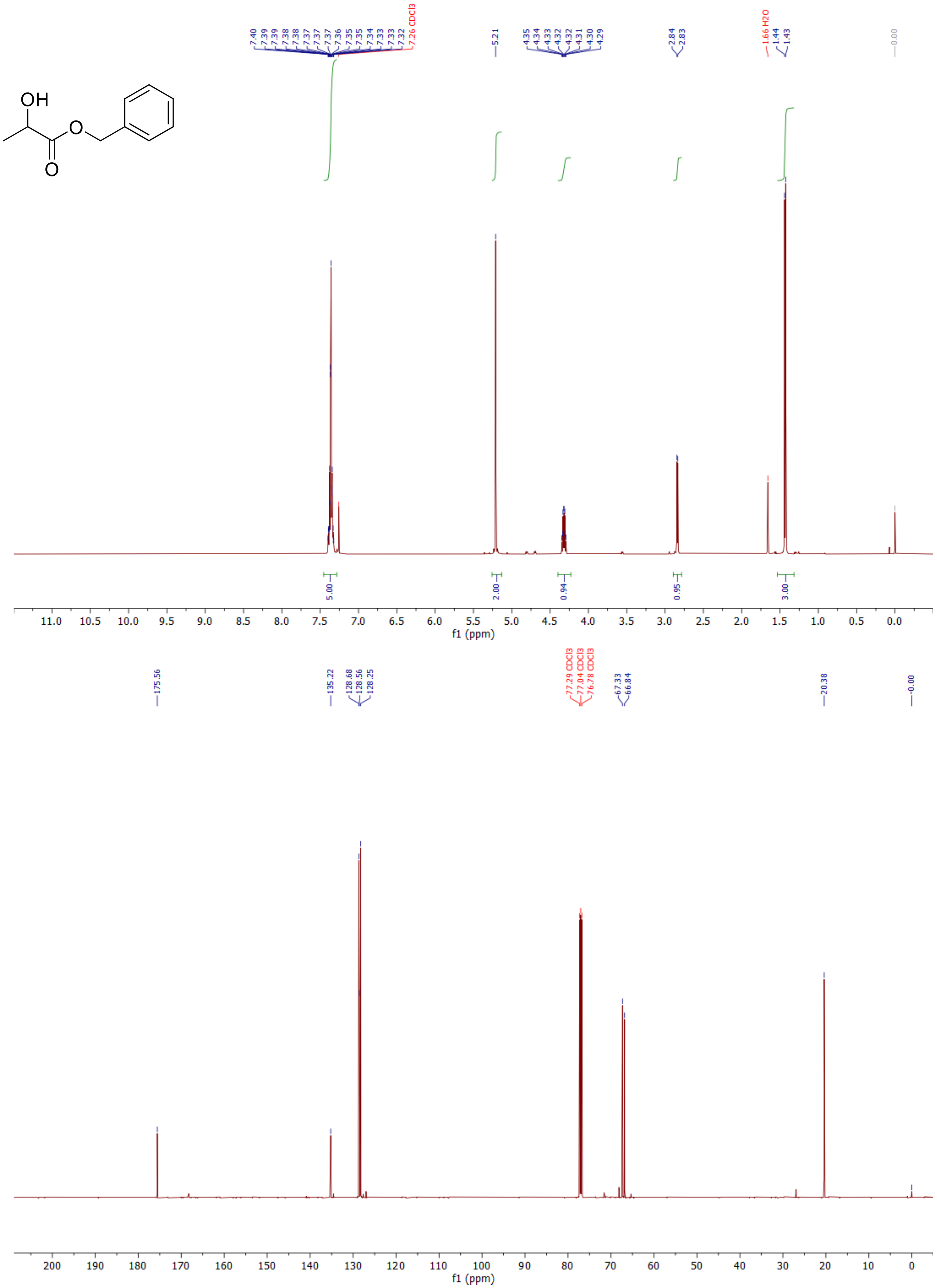

Figure S8: ^{1}H and ^{13}C NMR spectra of benzyl lactate **3a** in $CDCl_3$.

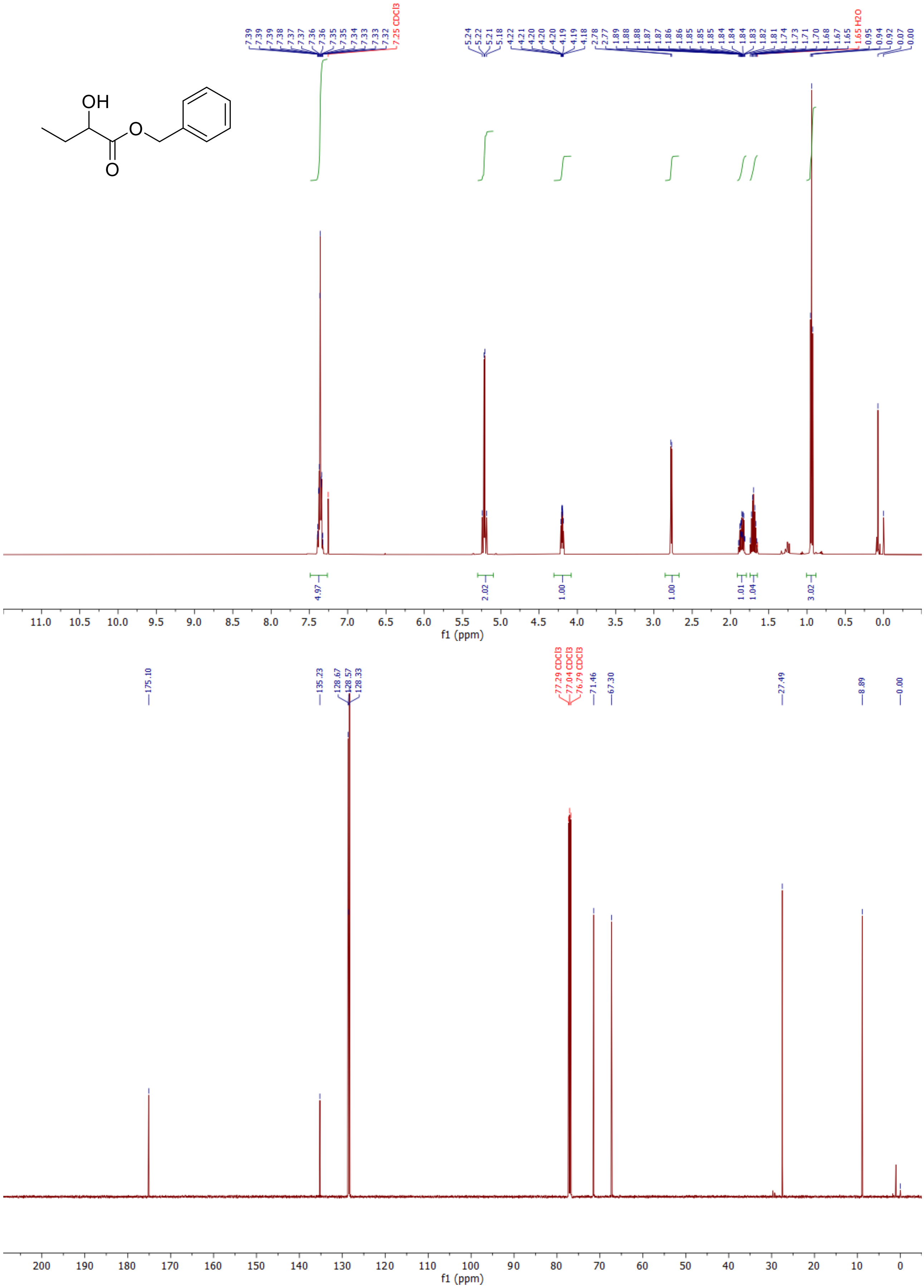

Figure S9: ^{1}H and ^{13}C NMR spectra of phenylmethyl 2-hydroxybutanoate **3c** in $CDCl_3$.

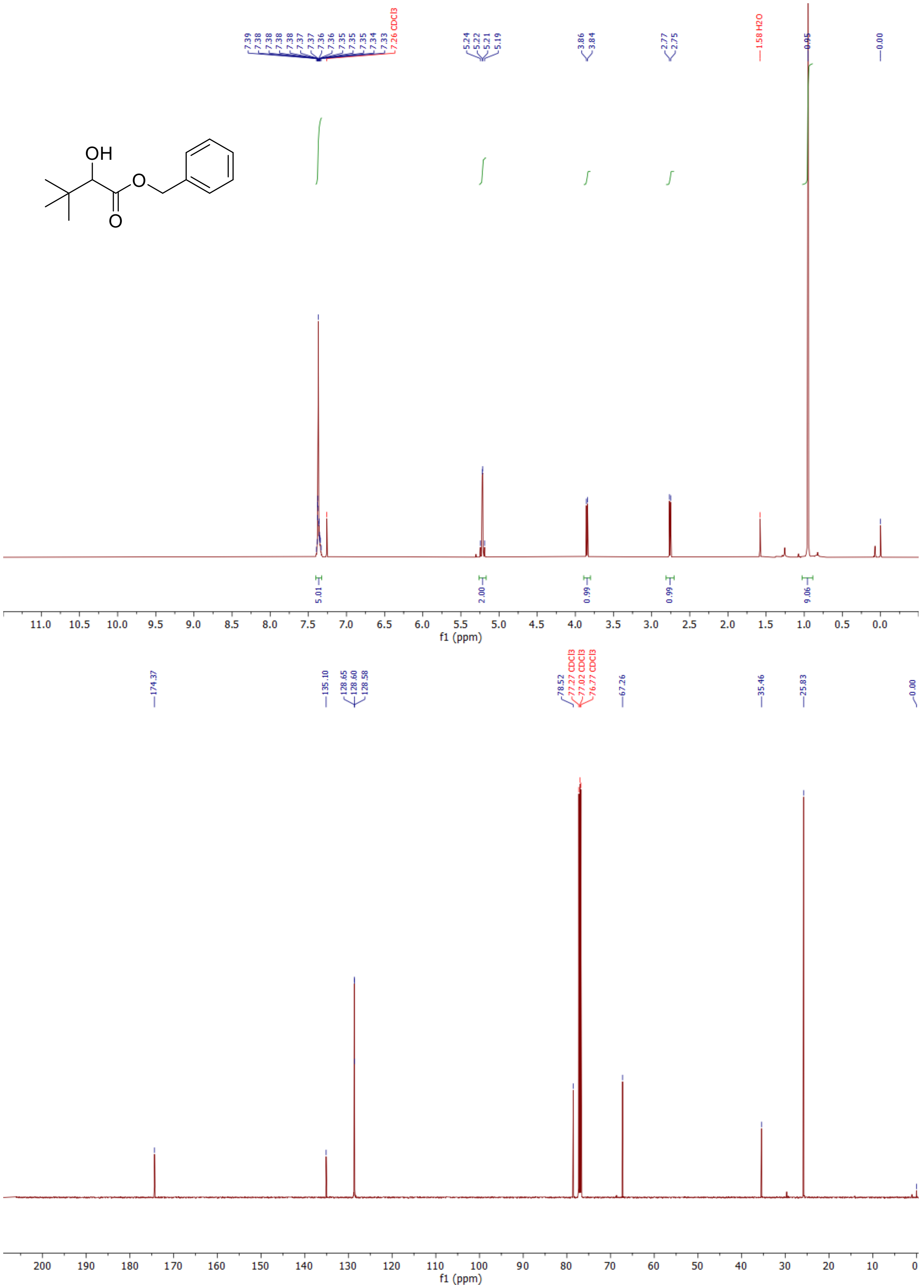

Figure S10: ^{1}H and ^{13}C NMR spectra of benzyl 2-hydroxy-3,3-dimethylbutanoate **3d** in $CDCl_3$.

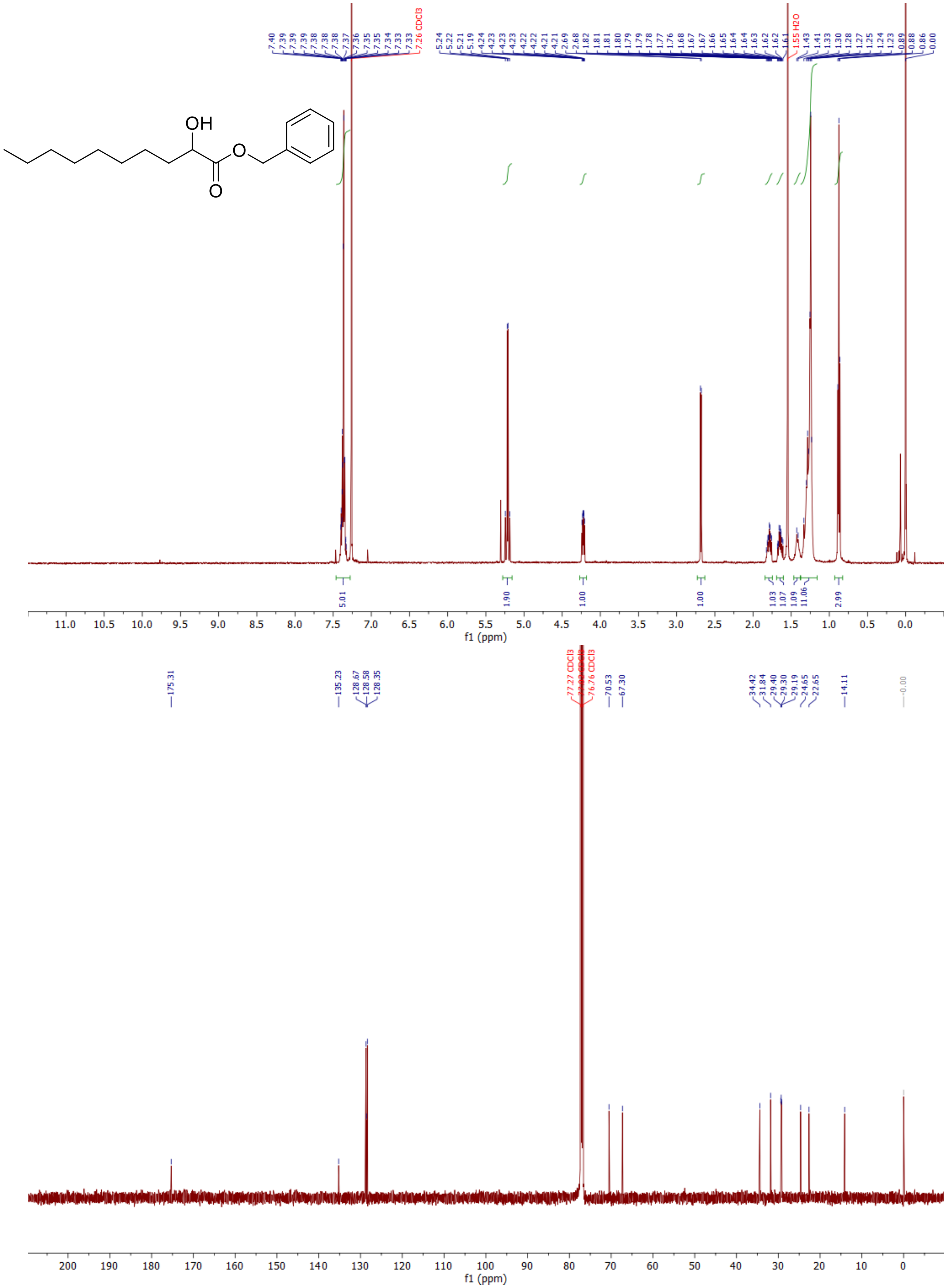

Figure S11: ^{1}H and ^{13}C NMR spectra of phenylmethyl 2-hydroxydecanoate **3e** in $CDCl_3$.

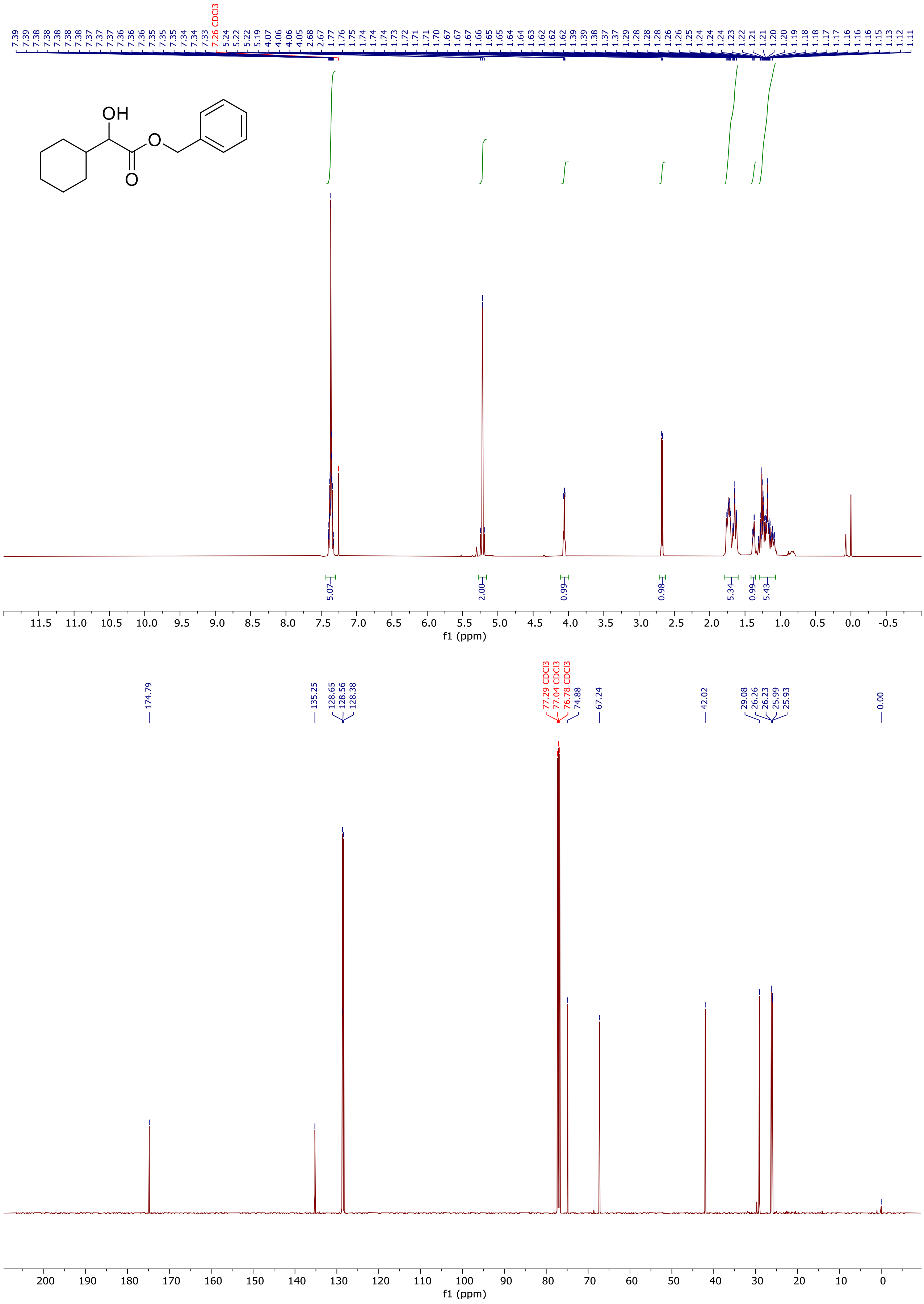

Figure S12: ^{1}H and ^{13}C NMR spectra of phenylmethyl α-hydroxycyclohexaneacetate **3f** in $CDCl_3$.

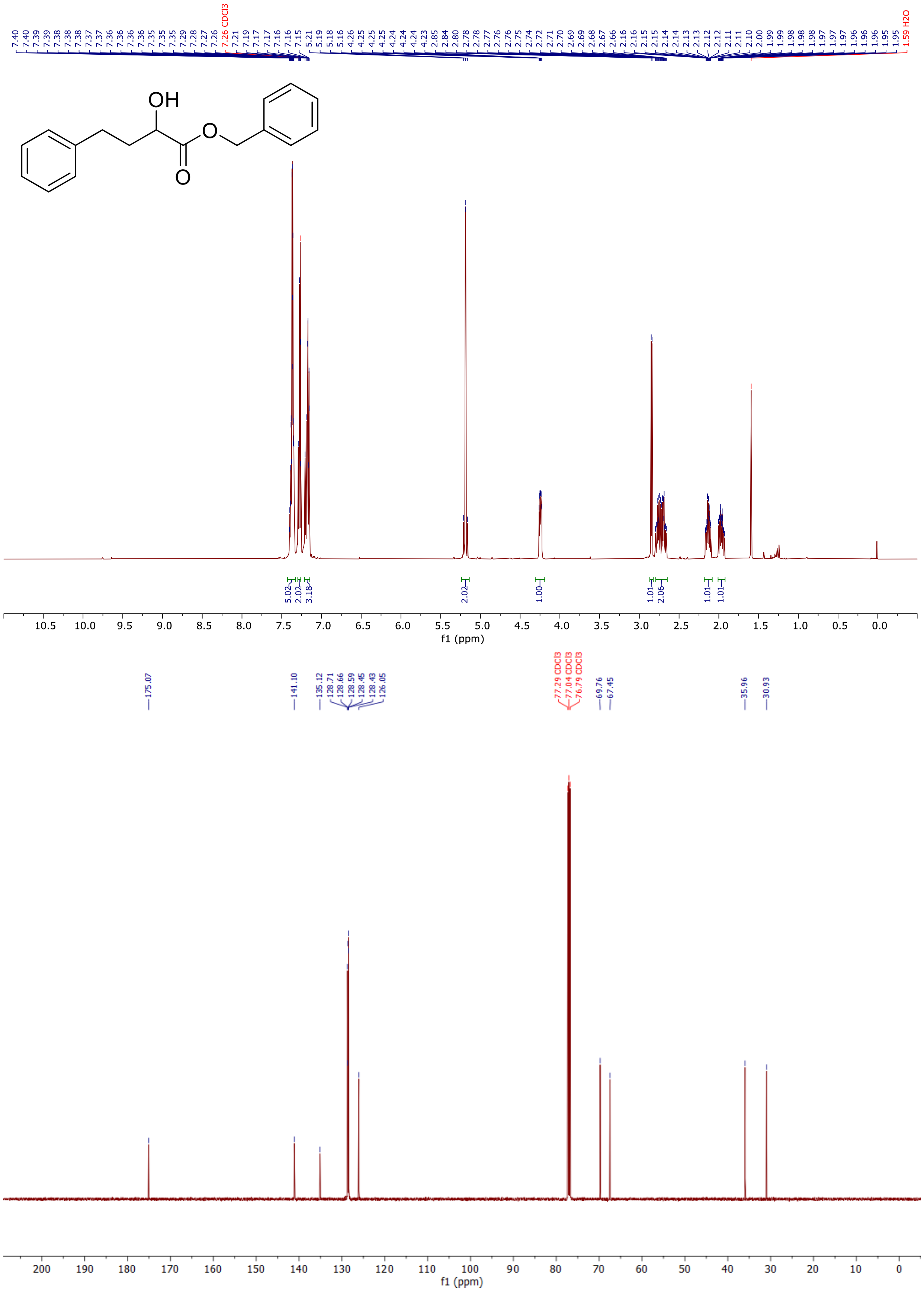

Figure S13: ^{1}H and ^{13}C NMR spectra of phenylmethyl α-hydroxybenzenebutanoate **3g** in $CDCl_3$.

10.Supporting Information References

1 M. Linden, S. Hofmann, A. Herman, N. Ehler, R. M. Bär and S. R. Waldvogel, *Angew. Chem. Int. Ed.*, 2023, **62**, e202214820.

2 P. J. H. Williams, G. A. Boustead, D. E. Heard, P. W. Seakins, A. R. Rickard and V. Chechik, *J. Am. Chem. Soc.*, 2022, **144**, 15969–15976.

3 T. Chatterjee, E. Boutin and M. Robert, *Dalton Trans.*, 2020, **49**, 4257–4265.

4 R. W. Clark, T. M. Deaton, Y. Zhang, M. I. Moore and S. L. Wiskur, *Org. Lett.*, 2013, **15**, 6132–6135.

5 P. S. Donnelly, A. J. North, N. C. Radjah, M. Ricca, A. Robertson, J. M. White and M. A. Rizzacasa, *Chem. Commun.*, 2019, **55**, 7699–7702.

6 Y. Zhang, X. Liu, L. Zhou, W. Wu, T. Huang, Y. Liao, L. Lin and X. Feng, *Chemistry*, 2014, **20**, 15884–15890.

7 S. S. Weng, H. C. Li and T. M. Yang, *RSC Adv.*, 2013, **3**, 1976–1986.

8 I. Shiina, K. Nakata, K. Ono, M. Sugimoto and A. Sekiguchi, *Chem. Eur. J.*, 2010, **16**, 167–172.

9 G. Silvestri, S. Gambino and G. Filardo, *Tetrahedron Lett.*, 1986, **27**, 3429–3430.

10 J. Seidler, A. Roth, L. Vieira and S. R. Waldvogel, *ACS Sustainable Chem. Eng.*, 2023, **11**, 390–398.
