# Supplementary material for: Synthesis of aliphatic α-hydroxy carboxylic acids via electrocarboxylation of aldehydes
Source: RSC Adv. 2025 Nov 21;15(53):45724–8. doi: 10.1039/d5ra07885g (PMC12637178; doi:10.1039/d5ra07885g)
Supplement: RA-015-D5RA07885G-s001 [file RA-015-D5RA07885G-s001.zip › NMRSpectra_Isolated_products/VO_benzyl_lactate/1/pdata/1/email_VO_benzyl_lactate_1_1.pdf]

F13+14

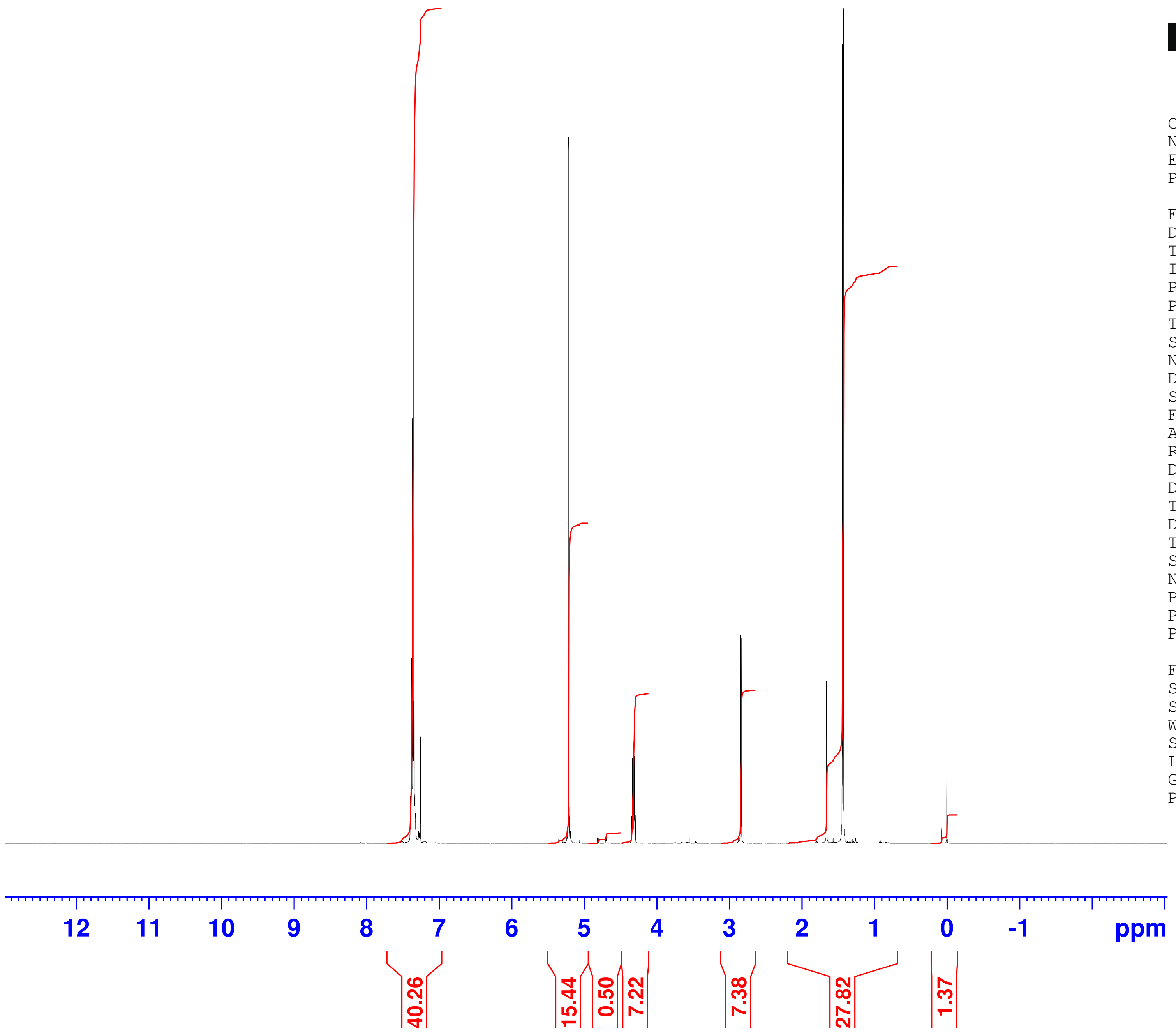

Current Data Parameters
NAME VO_benzyl_lactate
EXPNO 1
PROCNO 1

F2 - Acquisition Parameters
Date_ 20241025
Time 11.19 h
INSTRUM spect
PROBHD Z114229_0012 (
PULPROG zg30
TD 32768
SOLVENT CDCl3
NS 8
DS 4
SWH 8012.820 Hz
FIDRES 0.489064 Hz
AQ 2.0447233 sec
RG 97
DW 62.400 usec
DE 17.43 usec
TE 298.0 K
D1 10.00000000 sec
TD0 1
SFO1 500.1325007 MHz
NUC1 1H
P0 4.00 usec
P1 12.00 usec
PLW1 18.50000000 W

F2 - Processing parameters
SI 65536
SF 500.1300132 MHz
WDW EM
SSB 0
LB 0.30 Hz
GB 0
PC 1.00
