# Supplementary material for: Synthesis of aliphatic α-hydroxy carboxylic acids via electrocarboxylation of aldehydes
Source: RSC Adv. 2025 Nov 21;15(53):45724–8. doi: 10.1039/d5ra07885g (PMC12637178; doi:10.1039/d5ra07885g)
Supplement: RA-015-D5RA07885G-s001 [file RA-015-D5RA07885G-s001.zip › NMRSpectra_Isolated_products/VO_benzyl_lactate/5/pdata/1/email_VO_benzyl_lactate_5_1.pdf]

Final

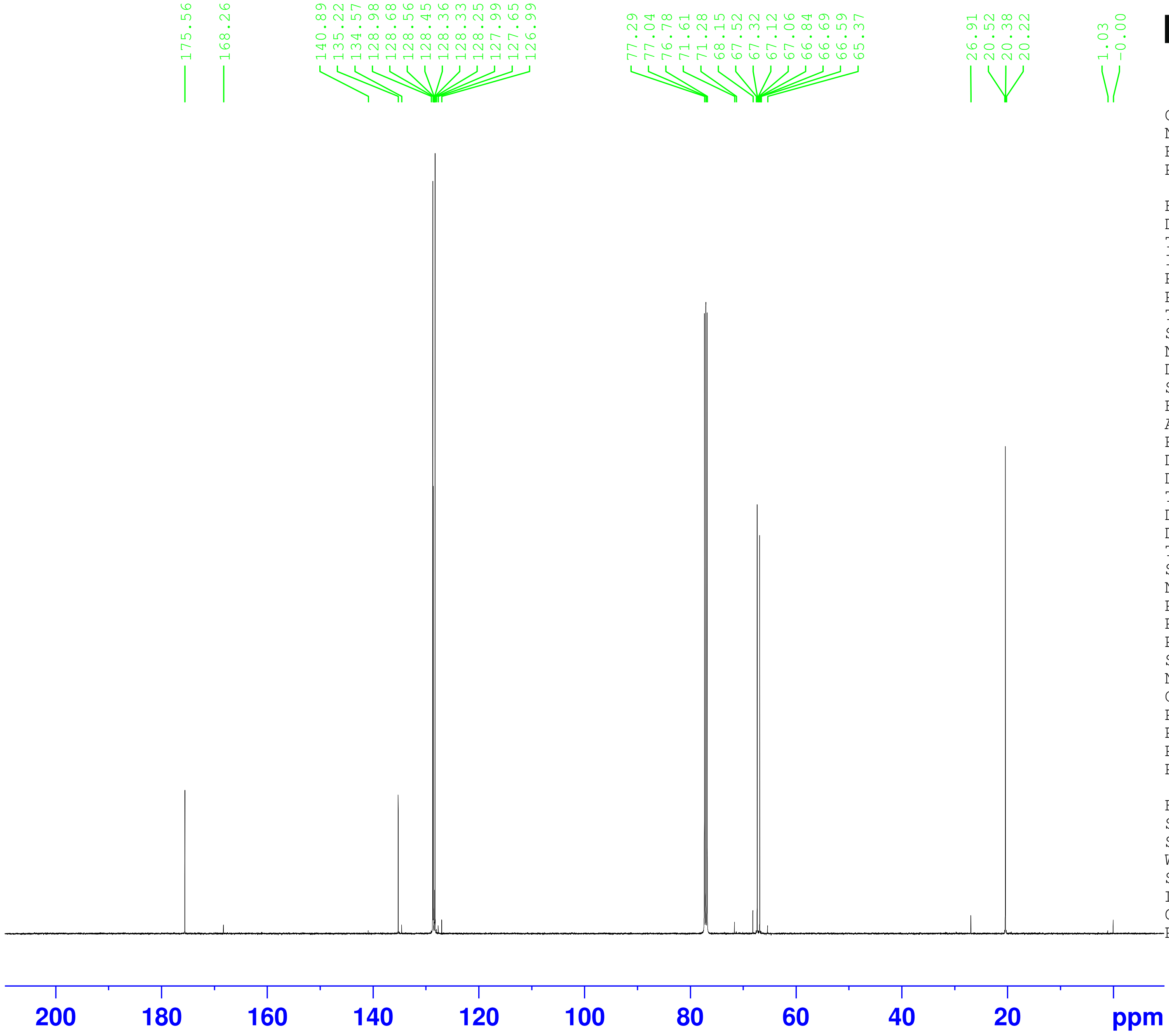

Current Data Parameters
NAME VO_benzyl_lactate
EXPNO 5
PROCNO 1

F2 - Acquisition Parameters
Date_ 20241028
Time 7.53 h
INSTRUM spect
PROBHD Z114229_0012 (
PULPROG zgpg30
TD 65536
SOLVENT CDCl3
NS 10000
DS 4
SWH 27573.529 Hz
FIDRES 0.841477 Hz
AQ 1.1883861 sec
RG 191.94
DW 18.133 usec
DE 6.50 usec
TE 298.0 K
D1 1.50000000 sec
D11 0.03000000 sec
TD0 1
SFO1 125.7703648 MHz
NUC1 13C
P0 2.83 usec
P1 8.50 usec
PLW1 114.00000000 W
SFO2 500.1325007 MHz
NUC2 1H
CPDPRG[2 waltz16
PCPD2 80.00 usec
PLW2 18.50000000 W
PLW12 0.41624999 W
PLW13 0.20937000 W

F2 - Processing parameters
SI 65536
SF 125.7577903 MHz
WDW EM
SSB 0
LB 1.00 Hz
GB 0
PC 1.40
